# Reduced Growth, Altered Gut Microbiome and Metabolite Profile, and Increased Chronic Kidney Disease Risk in Young Pigs Consuming a Diet Containing Highly Resistant Protein

**DOI:** 10.3389/fnut.2022.816749

**Published:** 2022-03-24

**Authors:** Margaret Murray, Melinda T. Coughlan, Anne Gibbon, Vinod Kumar, Francine Z. Marques, Sophie Selby-Pham, Matthew Snelson, Kirill Tsyganov, Gary Williamson, Trent M. Woodruff, Tong Wu, Louise E. Bennett

**Affiliations:** ^1^School of Chemistry, Monash University, Clayton, VIC, Australia; ^2^Department of Nutrition, Dietetics and Food, Monash University, Notting Hill, VIC, Australia; ^3^Department of Diabetes, Central Clinical School, Monash University, Melbourne, VIC, Australia; ^4^Baker Heart and Diabetes Institute, Melbourne, VIC, Australia; ^5^Monash Animal Research Platform, Monash University, Churchill, VIC, Australia; ^6^School of Biomedical Sciences, Faculty of Medicine, The University of Queensland, Brisbane, QLD, Australia; ^7^Hypertension Research Laboratory, School of Biological Sciences, Monash University, Clayton, VIC, Australia; ^8^Heart Failure Research Group, Baker Heart and Diabetes Institute, Melbourne, VIC, Australia; ^9^Bioinformatics Platform, Monash University, Clayton, VIC, Australia

**Keywords:** resistant protein, microbiome, metabolomics, protein fermentation, inflammation, kidney function

## Abstract

High-heat processed foods contain proteins that are partially resistant to enzymatic digestion and pass through to the colon. The fermentation of resistant proteins by gut microbes produces products that may contribute to chronic disease risk. This pilot study examined the effects of a resistant protein diet on growth, fecal microbiome, protein fermentation metabolites, and the biomarkers of health status in pigs as a model of human digestion and metabolism. Weanling pigs were fed with standard or resistant protein diets for 4 weeks. The resistant protein, approximately half as digestible as the standard protein, was designed to enter the colon for microbial fermentation. Fecal and blood samples were collected to assess the microbiome and circulating metabolites and biomarkers. The resistant protein diet group consumed less feed and grew to ~50% of the body mass of the standard diet group. The diets had unique effects on the fecal microbiome, as demonstrated by clustering in the principal coordinate analysis. There were 121 taxa that were significantly different between groups (adjusted-*p* < 0.05). Compared with control, plasma tri-methylamine-N-oxide, homocysteine, neopterin, and tyrosine were increased and plasma acetic acid was lowered following the resistant protein diet (all *p* < 0.05). Compared with control, estimated glomerular filtration rate (*p* < 0.01) and liver function marker aspartate aminotransferase (*p* < 0.05) were also lower following the resistant protein diet. A resistant protein diet shifted the composition of the fecal microbiome. The microbial fermentation of resistant protein affected the levels of circulating metabolites and the biomarkers of health status toward a profile indicative of increased inflammation and the risk of chronic kidney disease.

## Introduction

Protein is an essential macronutrient in the human diet needed for tissue growth and maintenance, the synthesis of enzymes and hormones that drive multiple integrated systems and functions, and as a source of energy (1). The recommended dietary protein intake for adults is 0.75–0.84 g/kg/day for women and men, respectively (2). Dietary protein is obtained from animal products, grains, cereals, legumes, nuts, and pulses (3). When consumed, protein undergoes enzymatic digestion and absorption in the small intestine followed by the microbial fermentation of unabsorbed portion in the large intestine. Enzymatic digestion by gastric and intestinal enzymes requires their interaction with specific amino acid recognition sequences and yields a mixed hydrolysates, such as large polypeptides, smaller peptides, and free amino acids (4). Progressive hydrolysis leads to the absorption of most dietary protein as amino acids in the jejunum (4). However, protein products, particularly larger peptides, that are resistant to enzymatic digestion (5, 6) are transported into the large intestine and undergo fermentation by the gut microbiota (4, 7).

Resistance to enzymatic digestion may result from certain conformations, natural ligands, and substituents that sterically protect otherwise digestible proteins (5). Alternately, resistance can arise from food processing. Heat-related chemical modifications that impose the loss of enzymatic recognition, such as the Maillard reaction, reduce digestibility (5, 6) and bioavailability of proteins (5, 8), and invoke microbial fermentation. Plant proteins are inherently less digestible than animal proteins, which is further attenuated by thermal processing in the presence of carbohydrates (Maillard chemistry), leading to the loss of digestibility (8). There are a number of converging factors that have increased exposure rates to less digestible, resistant forms of protein, in Western-style diets. These include the consumption of ultra-processed foods [such as, baked goods, reconstituted meat products, fast food, and many snack foods (9)], high-protein weight loss (such as, ketogenic) diets, the processed forms of infant formulae, and the popularity of “meat” substitutes made from processed plant proteins.

It is normal for a small proportion of dietary protein to be fermented in the large intestine. On average, 6–18 g of protein reaches the large intestine per day, a mixture of dietary and endogenous proteins (10) that is roughly proportional to total protein intake (11–13). However, process-modified resistant proteins increase the amount of dietary protein being fermented in the large intestine (14), which may result in health risks associated with the elevation of unhealthy protein fermentation products (7). Protein fermentation is associated with shifts in the composition, diversity, and/or relative abundance of gut microbial species, favoring nitrogen-utilizing, proteolytic microbes (11, 15–18). This poses a potential health risk when the microbiota are polarized toward nitrogen-utilization, and protein fermentation leads to the production and absorption of toxic levels of neuroactive, sulfide, aromatic and amine metabolites (7).

The metabolic pathways of protein fermentation yield multiple products capable of exerting independent effects on host tissues (7, 18, 19). Products include short- and branched-chain fatty acids, amines, ammonia, phenols, cresols, thiols, indoles, and sulfides, as well as neurotransmitters (e.g., gamma-aminobutyric acid (GABA), norepinephrine, dopamine, histamine, and serotonin) and other neuroactive compounds (e.g., tryptamine and phenethylamine) (7, 20–23). The normal levels of protein fermentation are of benefit to the host, for example, the fermentation process is required for the localized production of dopamine and serotonin that exert important signaling functions in the gut nervous system (20). However, we hypothesize that a diet high in resistant protein, and increased fermentation of resistant protein, contributes to poorer health status *via* several mechanisms related to gut microbiota composition, the production of neuroactive metabolites, and the promotion of inflammation. This pilot study aimed to examine the effects of a standard vs. highly resistant protein diet, fed at a normal protein level (21% w/w) on growth, gut microbiome, metabolomic profiles, and the biomarkers of disease risk, to identify the outcomes of interest for further study. Here, pigs are used as an appropriate animal model for human digestion and metabolism.

## Materials and Methods

### Animals

**Figure 1:**
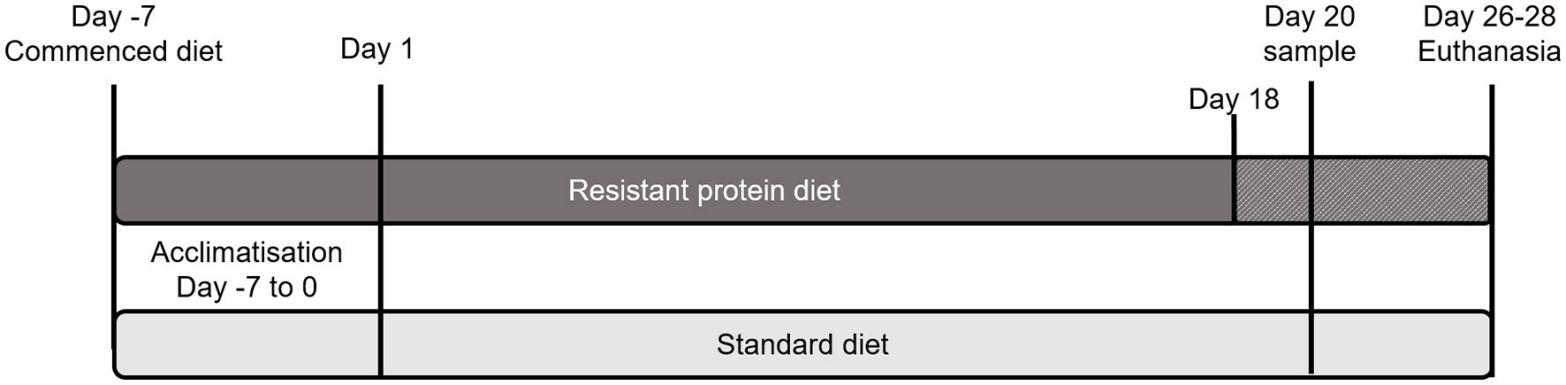
Schematic of the study design showing the period of acclimatisation and the schedule of standard and resistant protein diets, including supplementation of resistant protein with the standard diet from Day 18 and sampling on day 20.

Ethical approval for the project was granted by the Monash Animal Research Platform-1 Animal Ethics Committee (Approval No. 17533). This work was carried out in accordance with the Australian code for the care and use of animals for scientific purposes (24) and is reported in accordance with the ARRIVE guidelines (25). A pig model of human digestion and metabolism was used for this study as pigs have a similar diet (omnivorous) and digestive system to humans. The study involved two groups of four male weanling pigs (Large White cross Landrace). One extra animal for each group was kept in the circumstance where an animal could not continue the project due to ill health or injury, making a total of five pigs per group. The pigs were 3–4 weeks old and newly weaned on the commencement of study acclimatization period. Each group was from a single litter and was housed together for the study duration (12 h natural light/dark cycle, ambient temperature 15–20°C, with straw bedding). One group was fed a standard diet (control) and the other group was fed a resistant protein diet (intervention), each containing 21% w/w protein. Feed was administered in controlled quantities to meet 100% of the pigs' energy requirements (~530 kJ digestible energy/kg bodyweight/day), and water was given *ad libitum*. Pigs were acclimatized on their respective diets for 7 days and monitored daily throughout this period. Following acclimatization, individual feeding was conducted two times daily in metabolic cages throughout the study, and pigs were retained in the cages on the morning of day 20 until a fecal sample was produced (Figure 1). For the duration of the study, pigs were monitored 3 times per week for condition; feed intake was recorded on a daily basis and body weight (to nearest 0.5 kg) was recorded two times per week. The resistant protein diet group had lower feed intake than the standard diet group, thus their diet was substituted by 25% with the standard diet from day 18 onwards.

### Sample Collection, Biochemistry, and Hematology

Fecal samples, collected on day 20, were snap frozen (dry ice) and stored at −80°C. Venous blood samples were collected as a terminal sample immediately prior to euthanasia (6–8 days after day 20 sample collection). Animals were anesthetised *via* the mask inhalation of Sevoflurane and blood samples were collected from the anterior vena cava into plain tubes (10 ml) for biochemistry, fluoride oxide tubes for glucose evaluation, and ethylenediaminetetraacetic acid (EDTA) tubes for hematology (10 ml). Samples were stored at 4°C before transport and analysis for general hematology and biochemistry markers (Gribbles Veterinary Pathology, Glenside, South Australia), such as plasma alkaline phosphatase (ALP), gamma-glutamyl transferase (GGT), aspartate aminotransferase (AST), urea, and creatinine. Kidney function marker, estimated glomerular filtration rate (eGFR), was calculated from body weight and the measures of plasma urea and creatinine, using a formula appropriate for swine (26).

### Characterization of Diets

The standard and resistant protein diets were matched for energy, macro-, and micro-nutrient composition (Supplementary Table 1). The standard diet was a laboratory pig weaner diet (SF18-148 Specialty Feeds, Glen Forrest, WA) containing wheat, barley, lupins, soya meal, calcium carbonate, salt, dicalcium phosphate, lysine, and a vitamin and trace mineral premix; digestible energy 13.3 kJ/g. The resistant protein diet was a skim milk powder pig weaner diet (SF18-147 Specialty Feeds) containing barley, skim milk powder (supplied by Tatura Milk Industries, Tatura, Victoria), soya meal, canola meal, calcium carbonate, salt, dicalcium phosphate, and a vitamin and trace mineral premix; energy 13.3 kJ/g. The resistant protein diet was treated by autoclave heating (15 h at 70°C followed by 20 min at 121°C) to drive the Maillard chemistry of proteins and carbohydrates and confer digestive resistant status to the protein. The resistant protein was expected to have low enzymatic digestibility and to preferentially undergo colonic fermentation. The effect of heat treatment on micronutrients was measured by the analysis of thiamine (vitamin B_1_) content (PathWest Laboratory Medicine, Nedlands, WA). The heat treatment of the resistant protein diet was designed to simulate the high heat processing that many ultra-processed food products undergo, to create a (somewhat extreme) model for a diet high in ultra-processed foods and resistant proteins.

The *in vitro* digestibility of proteins in the standard and resistant protein diets was measured by the method reported in Wu et al. (27), which simulates adult digestion conditions using gastric and intestinal enzymes and a standardized ratio of enzymes to protein nitrogen. Briefly, the method involved gastric digestion using pepsin (60 min, 0.5 ml, and 7 mg/ml) followed by duodenal digestion using a pancreatin-bile solution (180 min, 10 mg/ml pancreatin, and 60 mg/ml bile salt). Dried feed samples were dispersed in simulated gastric buffer (0.15 M sodium chloride, pH 2.5) at 1.25 mg total nitrogen/ml. Samples, such as simulated gastric buffer controls (no test protein), were preheated to 37°C and pepsin was added to initiate digestion. Sampling (125 μl) was conducted every 15 min for 60 min, with the inactivation of enzymes achieved by the addition of an equal volume of 0.25 M NaOH solution. After 60 min, the pH was adjusted to 7.5 (1 M sodium hydroxide) and pancreatin-bile solution in simulated duodenal buffer (150 mM sodium chloride, 1.0 mM sodium bicarbonate, and pH 7.5) was added. The simulated duodenal digestion proceeded for 180 min, with samples taken every 30 min (125 μl), followed by the chemical inactivation of enzyme. All samples were stored at −20°C until analysis of the degree of hydrolysis using derivatisation with o-phthaldialdehyde, monitored by a microplate reader (Excitation = 340 nm and Emission = 450 nm) (27). A standard curve was prepared using L-serine and the results of duplicate sample analysis are reported as the equivalents of L-serine.

### Analysis of Fecal Microbiome

Fecal samples, collected on day 20, from three pigs in each group were sequenced for diversity profiling to compare the microbiome between the two groups. Polymerase chain reaction (PCR) amplification and sequencing were performed by the Australian Genome Research Facility (Melbourne, VIC). PCR amplicons were generated using the 341F and 806R primers to amplify the V3-V4 region of the 16S gene. Thermocycling was completed with an Applied Biosystem 384 Veriti and using Platinum SuperFi mastermix (Life Technologies, Australia) for the primary PCR. The first stage PCR was cleaned using magnetic beads, and samples were visualized on 2% Sybr Egel (Thermo-Fisher). A secondary PCR to index the amplicons was performed with TaKaRa Taq DNA Polymerase (Clontech). The resulting amplicons were cleaned again using magnetic beads, quantified by fluorometry (Promega Quantifluor) and normalized. The eqimolar pool was cleaned a final time using magnetic beads to concentrate the pool and then measured using a High-Sensitivity D1000 Tape on an Agilent 2200 TapeStation. The pool was diluted to 5 nM and molarity was confirmed again using a High-Sensitivity D1000 Tape. This was followed by sequencing on an Illumina MiSeq (San Diego, CA, USA) with a V3, 600 cycle kit (2 × 300 base pairs paired-end).

### 16S rRNA Gene Bioinformatics Analyses

The DADA2 R package (28) was used for reads pre-processing and denosing, and the phyloseq R package for further analyses (29). For FASTQ trimming and filtering, “filterAndTrim” function was used to form the packages, setting “trimLeft” option to 20 bases for both R1 and R2 reads to remove adaptor sequences at the 5 prime of the reads, and “truncLen” option to 267 and 222 for R1 and R2, respectively, to truncate reads length at the 3 prime of the reads due to low base quality, “maxEE” was set to 2 for both R1 and R2 reads to remove reads that have too high expected error. In addition, auxiliary functions were used to dereplicate samples, to merge paired-end reads, and to estimate and correct for read errors, before applying the “Dada2” function to pick amplicon sequence variants (ASVs) with “pool” option set, which aggregates all samples together for the greater sensitivity of ASV selection. After the filtering, ASV selection and chimeric reads removal, there were between 21,875 and 51,408 unique reads that formed 2,916 unique ASVs. For *de novo* ASVs annotation, a greengenes classifier (version gg_13_8_train_set_97.fa.gz) with “assignTaxonomy” function was used. In some of the downstream analyses, ASVs were collapsed into corresponding phylum or genus bins. Additionally, a phylogenetic tree was generated using phangorn R package (30), “optimal.plm” function using GTR model, and nearest neighbor interchange rearrangement, to perform phylogenetic tree-based filtering, to remove ASVs that appeared outliers on the tree and were not of bacterial 16S rRNA origin, according to the BLAST cross referencing.

To understand individual sample α-diversity, data were analyzed using Chao1, Shannon, and Simpson's indices and plots were generated with “plot_richness” function from phyloseq package. To assess β-diversity, datasets were visualized using unweighted UniFrac distance with “ordinate” function and visualized with “plot_ordination” function. Moreover, we used the same “ordinate” and “plot_ordination” function, but with two different distances types, Bray–Curtis dissimilarity and weighted UniFrac, as a function of either diets or treatment groups. Both metrics gave very similar results estimating β-diversity. Additionally, to test the significance of the separation of the clusters between diet groups, we used a permutational multivariate analysis of variance (PERMANOVA) test, implemented in “Adonis” function, from vegan R package (31, 32) on weighted UniFrac distances with default, 999 permutation to form pseudo F-distribution.

For differential taxa occurrence, we used DESeq2 R package (33). Raw taxa counts were normalized for library size with counts per million and we used “DESeqDataSetFromMatrix” function to form a DESeq object and fitted with emodels with “DESeq” function using the Wald test and parametric fit type. Significance was determined as false discovery rate (FDR) <0.05 (34).

### Analysis of Metabolites in Plasma

The quantification of metabolites was undertaken using an Agilent 1200 series high-performance liquid chromatography (HPLC) system (Agilent 255 Technologies) coupled to tandem mass spectrometry (API 3200, AB SCIEX) with electrospray ionization following previously validated protocols (35–40). All study samples were processed, run, and analyzed as a single batch. For short- and medium-chain fatty acids and ketosis markers, a chromatographic separation of processed plasma samples was conducted using a Kinetex EVO C18 analytical column (100 × 2.1 mm, 100 Å, 5 μm, Phenomenex Inc., CA, USA) under binary gradient conditions (mobile phase A: 0.1% formic acid in milliQ water containing 10 mM ammonium formate, pH 3, and mobile phase B: 0.1% formic acid in 9:1 methanol:isopropanol solution), as derivatives of benzyloxy-amide (40–42), and 2-ethyl butyric acid as internal standard. For amino acids and neurotransmitters, chromatographic separation was achieved by Luna Omega Polar C18 analytical column (100 × 2.1 mm, 100 Å, 3 μm, Phenomenex Inc., CA, USA) under binary gradient conditions using mobile phase A (as above) and mobile phase B (0.1% formic acid in acetonitrile). Plasma samples with D9-Choline as internal standard were processed and derivatised as benzoyl chloride derivatives using benzoyl chloride in acetonitrile buffered at alkaline pH with volatile ammonium carbonate for MS compatibility (43). For all other metabolites, chromatographic separation was implemented by Ascentis Express HILIC column (150 × 2.1 mm, 100 Å, 2.7 μm, Supelco) under binary gradient conditions using mobile phase A (as above) and mobile phase B (as above) along with D9-Choline and 1-(4-fluorobenzyl)-5-oxoproline as internal standards for positive and negative mode, respectively. All experimental data processing and analysis were performed by Analyst (AB SCIEX, USA, version 1.6.2) and Multiquant software (AB SCIEX, USA, version 2.0).

### Statistical Analysis

This was a pilot study, designed to discover the outcomes of interest in relation to high resistant protein diets, and therefore was not powered based on any one outcome measure. Data were tested for normality using Shapiro–Wilk test. Data for eGFR and liver function markers were analyzed using an independent *t*-test. Differences between the groups for feed intake, body mass, and thiamine intake on each day were assessed using the Mann–Whitney *U*-test for independent groups, with a significance threshold of *p* < 0.05. Data from targeted metabolomics analysis were mean-centered and divided by the standard deviation (SD) of each analyte. Significance was identified by a fold change threshold of 2 and a FDR (q) of 0.1, using Metaboanalyst (version 5.0).

## Results

### Protein Digestibility

**Figure 2:**
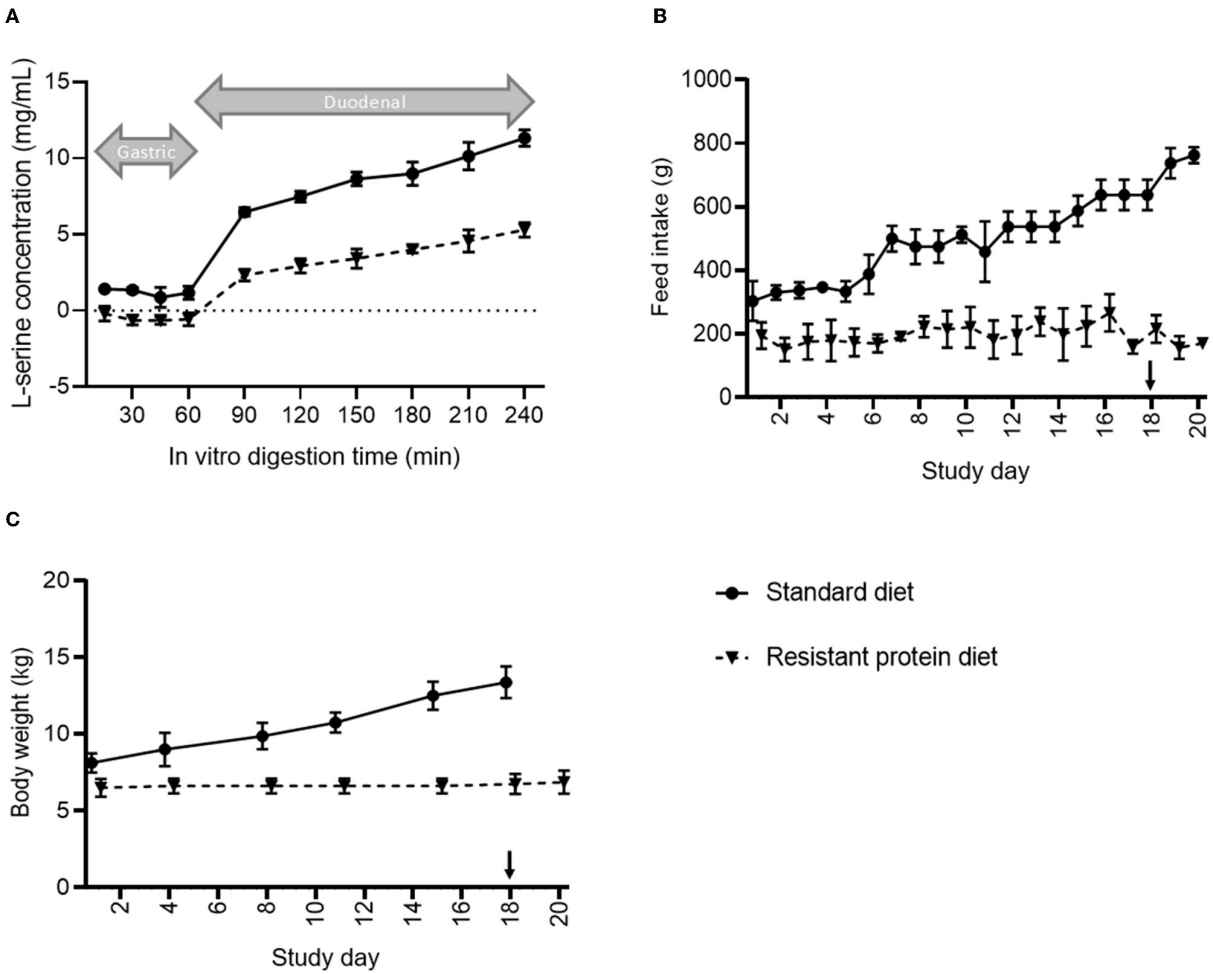
Comparison of **(A)** *in vitro* digestibility, **(B)** average daily feed consumption, and **(C)** changes in the body mass of pigs fed standard vs. resistant protein diets, where ‘↓' indicates where the resistant protein diet was supplemented with 25% of the standard diet. Results represent the mean and SD of data for *n* = 4 pigs per group. From day 1 onwards, feed intake and body weight were significantly different between groups (*p* < 0.05).

Following sequential *in vitro* treatment with gastric and intestinal enzymes, the digestibility of the resistant protein diet was found to be approximately 50% that of the standard diet (Figure 2A). This indicates that the upper gut bioavailability of proteins in the resistant protein diet was lower than that for proteins in the standard diet.

### Effect of Diets on Pig Health

The mean bodyweights for the pigs in the resistant protein diet group (mean ± SD, 5.8 ± 0.7 kg) and standard diet group (8.3 ± 1.4 kg) were significantly different at baseline (*p* < 0.01), and their feed intakes and bodyweights were significantly different for the duration of the study (Figure 2). Although the difference in baseline mean bodyweights between the groups may have contributed to the differences in subsequent growth, it was clear that the resistant protein diet group ate significantly less of the resistant protein feed from day 1 onwards (Figure 2B, *p* < 0.05), which can account for their lower rate of growth (Figure 2C). The reduced feed intake may also have indirectly affected other markers assessed. Due to the lack of growth and initial signs of condition loss among the resistant protein diet pigs, the resistant protein feed was supplemented with 25% of the standard diet from day 18 onwards. The thiamine (vitamin B_1_) content was significantly reduced in the resistant protein diet (40.5 vs. 111 μg/g, resistant vs. standard protein) indicating nutrient loss associated with autoclaving. The feed intakes of the resistant protein diet group produced a nutritional deficiency in thiamine (meeting 45–85% of recommended daily intake).

**Figure 3:**
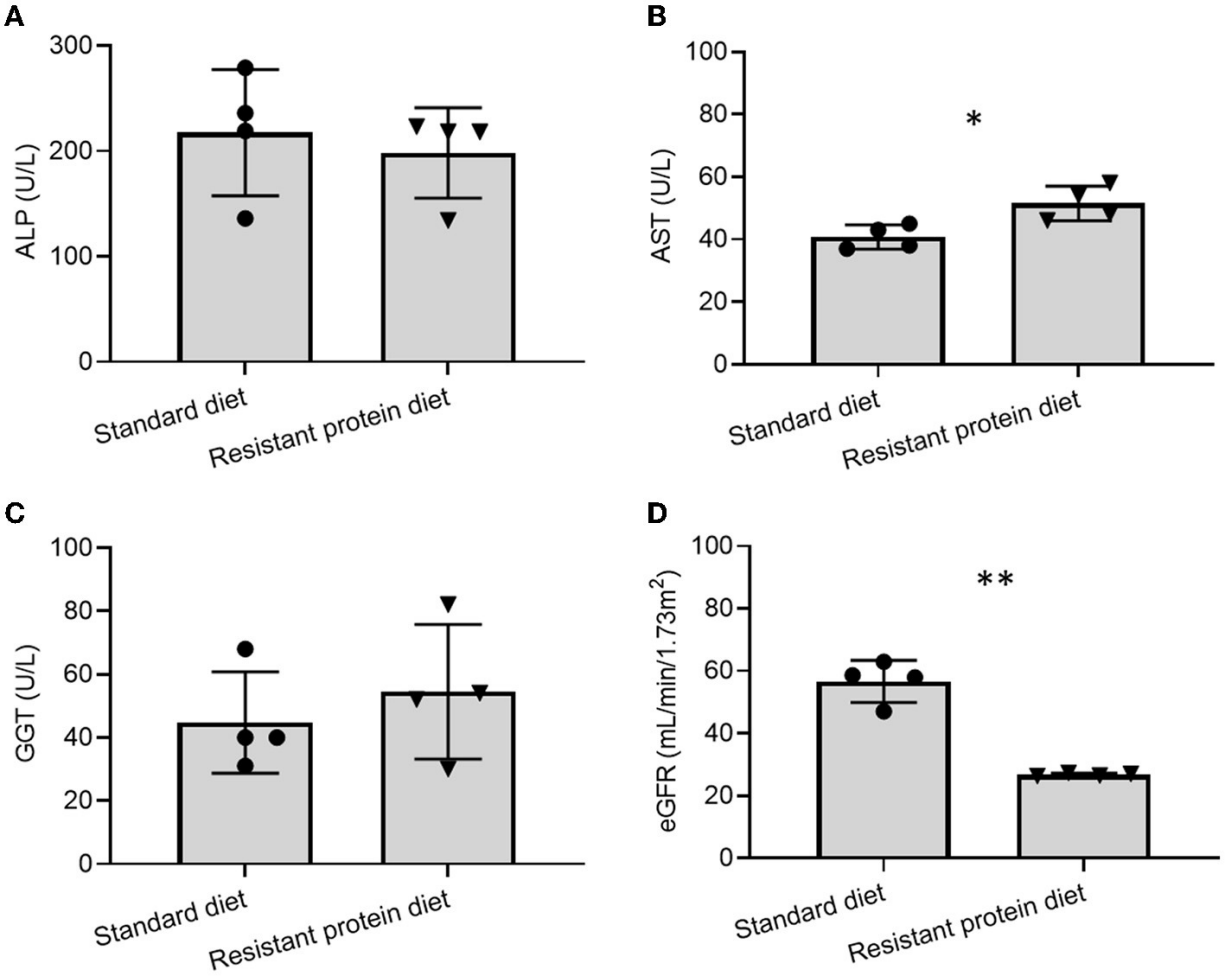
Comparison of effects of standard and resistant proteins diets on the biomarkers of liver and renal function, showing results for **(A)** alkaline phosphatase (ALP), **(B)** aspartate aminotransferase (AST), **(C)** gamma-glutamyl transferase (GGT), and **(D)** estimated glomerular filtration rate (eGFR). Results represent the mean and SD of data, with dots indicating individual data points. Statistical difference between groups is designated as follows: *p = 0.019, **p < 0.001.

The biomarkers of liver function were similar between the two groups (ALP and GGT *p* > 0.05, Figures 3A,C). Compared with the standard diet group, the resistant protein diet group had slightly increased AST (*p* = 0.019, Figure 3B), which could suggest a worsening liver function in that group, however, the levels of AST in both groups were within the reference range of 5–60 U/L. The kidney function of the resistant protein diet group, indicated by eGFR, was significantly lower than the standard diet group (Figure 3D, *p* = 0.001). Other hematology and biochemistry markers indicated similar functional and health status between the two groups (Supplementary Table 2).

### Effect of Diets on Pig Fecal Microbiome

**Figure 4:**
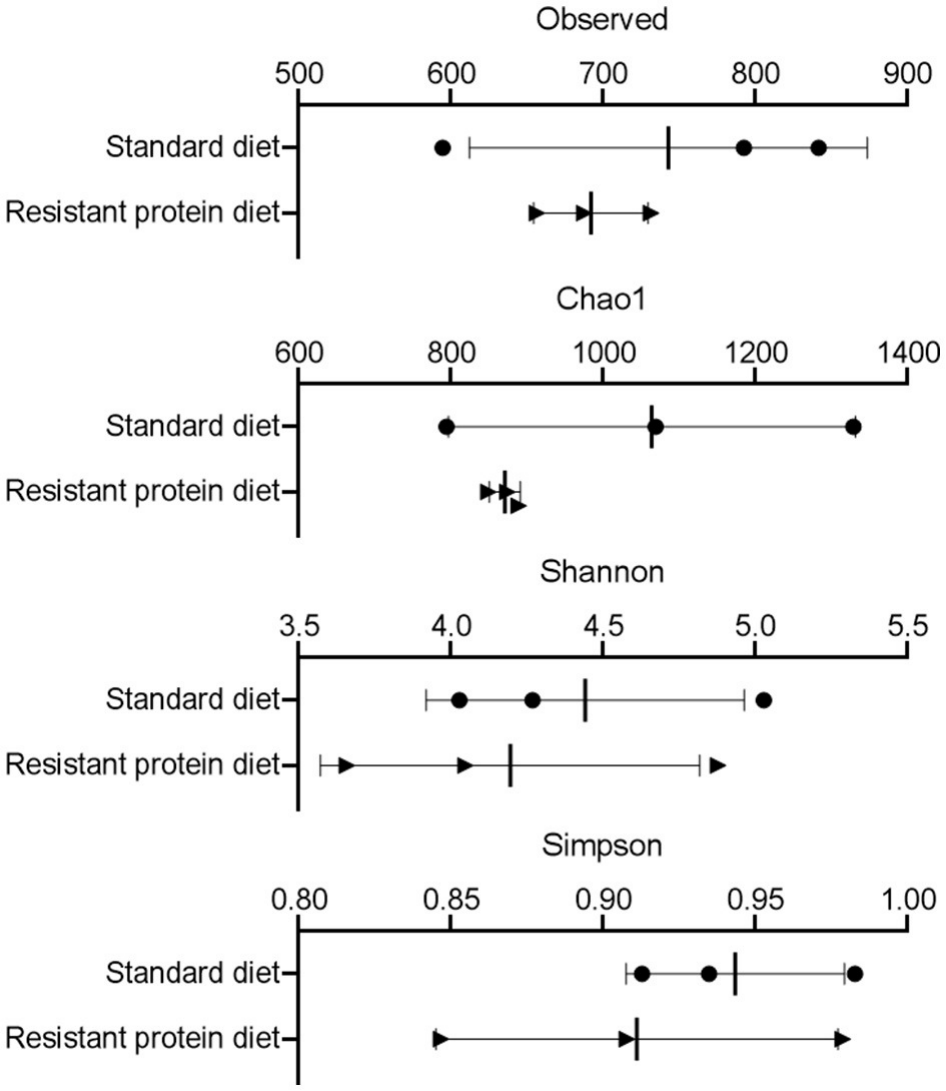
The effects of standard and resistant protein diet on pig microbiota at day 20 showing effects on scores of α-diversity by measures of Observed taxa, Chao1, Shannon, and Simpson indices (*n* = 3 pigs per treatment). Results represent the mean and SD of data, with dots indicating individual data points. There was no significant difference between diets on α diversity (all *p* > 0.05).

**Figure 5:**
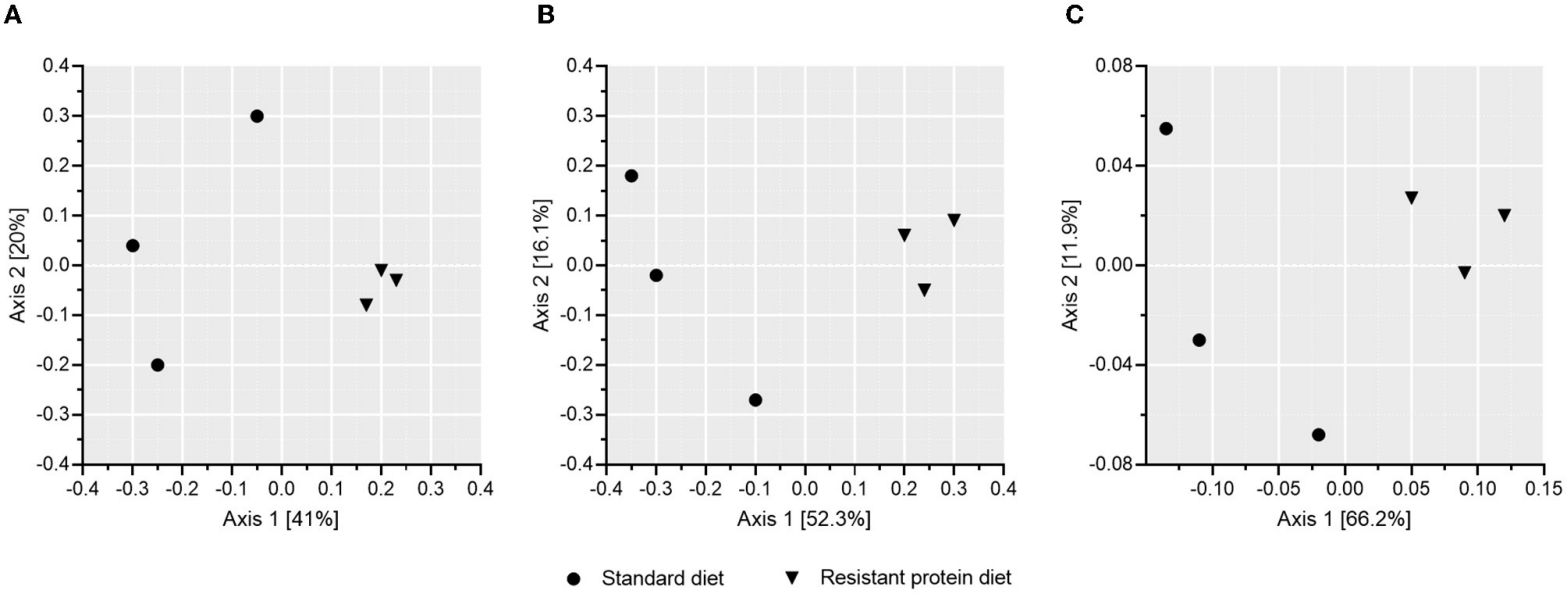
The effects of standard and resistant protein diet on pig microbiota at day 20 showing effects on β-diversity according to mean scores of **(A)** unweighted Unifrac, **(B)** Bray–Curtis, and **(C)** weighted UniFrac analyses (presence/abundance of taxa), which indicate significant differentiation of the microbial composition due to diets (*n* = 3 pigs per treatment, all *p* < 0.05).

The effect of standard and resistant protein diets on fecal microbiome diversity and composition were assessed. The α-diversity indices, such as Observed, Chao1, Shannon, and Simpson representations, each indicated equivalent numbers of taxa and no differences in diversity or richness between the diets (Figure 4, all *p* > 0.05). However, the difference in the microbial composition between the groups was evident in the analyses of β-diversity (Figure 5), where there was a large separation between the groups in unweighted and weighted UniFrac analyses as well as Bray-Curtis distance (all *p* < 0.05). In the unweighted UniFrac principal coordinate analysis plot, the first dimension explained 41% of the total variability in the data, suggesting that different bacteria were present according to diet (Figure 5A). In the Bray–Curtis (Figure 5B) and weighted UniFrac (Figure 5C) principal coordinate analysis plot, the first dimension explained 52.3–66.2% of the total variability in the data, respectively, suggesting that the abundance of certain bacteria differed between the groups.

**Figure 6:**
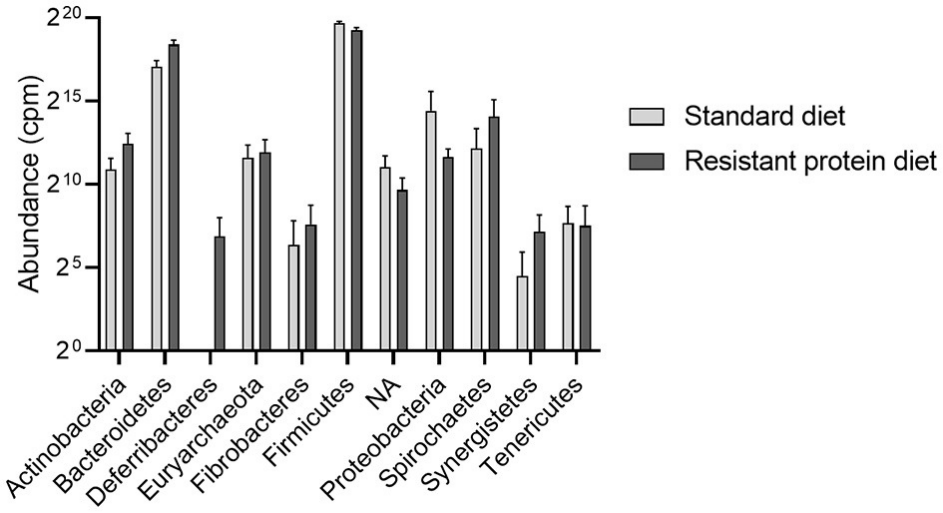
The effects of standard and resistant protein diet on the distribution of phylum abundance in pig faecal microbiota at day 20 showing the relative abundance of the dominant phyla. Data represent the mean and SD (*n* = 3 per group). There were no significant differences between groups for any phyla. NA, phylum unspecified; cpm, counts per million.

The most abundant taxa were clearly different between the two diet groups, with the standard diet group being dominated by bacteria from the phylum Firmicutes, such as *Kandleria, Butyrivibrio, Streptococcus, Catenibacterium*, and *Megasphaera* genus. In contrast, the resistant protein diet produced a high prevalence of *Bacteroidetes* phylum, such as *Prevotella*, and Firmicutes phylum, such as *Lactobacillus* sp and *Blautia* genus. Indeed, when a differential taxa occurrence analysis was performed, there were 121 ASVs that were significantly different between diets, after adjustment for multiple comparisons (*q* < 0.05, Supplementary Table 3). These findings were validated by an independent linear discriminant analysis. Both groups shared similar prevalence of the phyla Firmicutes, *Bacteroidetes*, Euryarchaeota, *Actinobacteria, Proteobacteria, Spirochaetes*, and *Tenericutes*. Whereas, WPS-2, TM7, and *Cyanobacteria* (although this may represent ingested chloroplasts, contamination or related phylum, such as *Melainabacteria*) were only observed in the standard diet group (Figure 6).

### Effect of Diets on Plasma Metabolites

**Figure 7:**
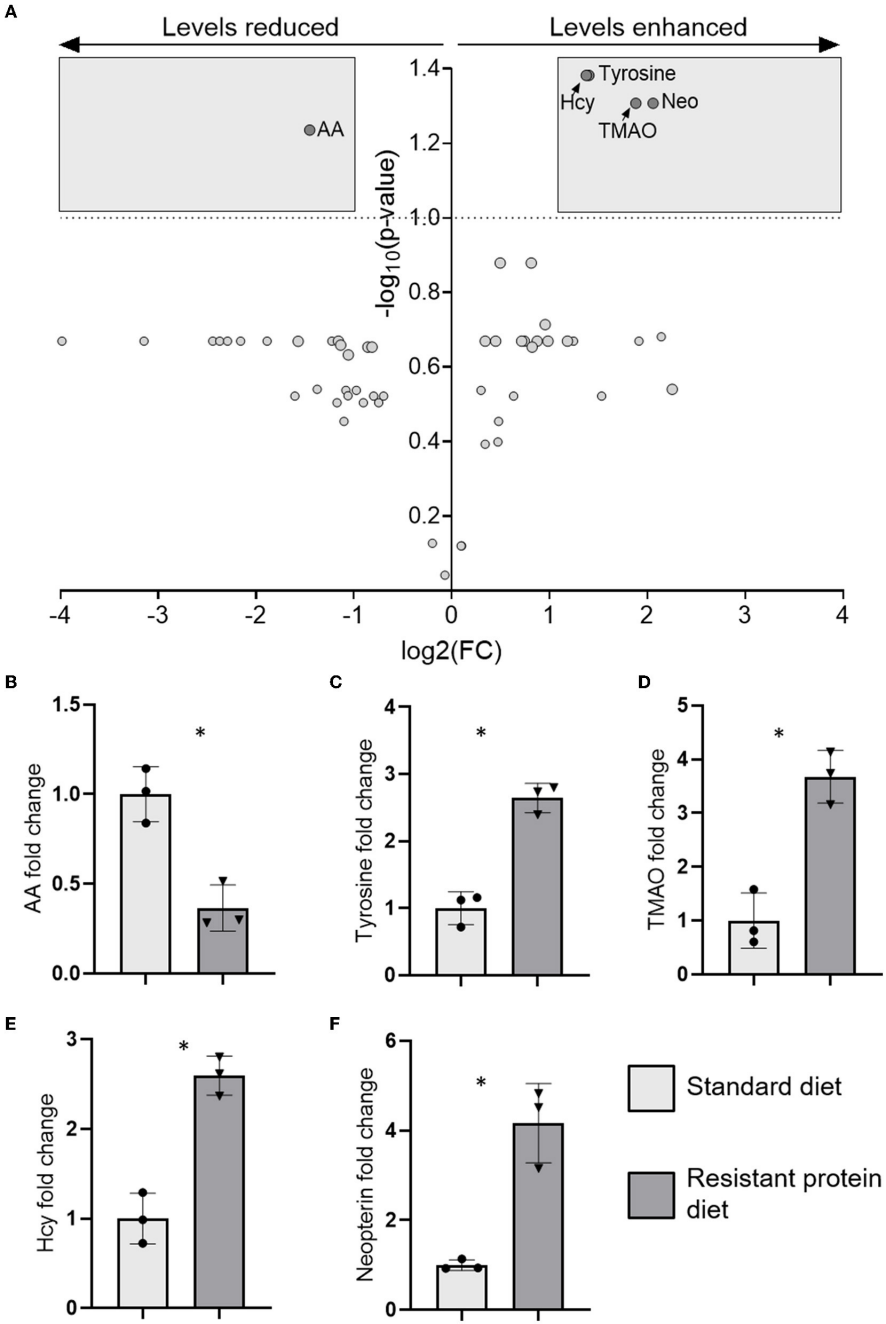
The results of metabolomics analysis in plasma showing **(A)** volcano plot of five significantly differentiated metabolites after applying a fold-change threshold of 2 and false discovery rate (FDR) (q) of 0.1, and the fold change of those individual metabolites **(B)** acetic acid (AA), **(C)** tyrosine, **(D)** tri-methylamine-N-oxide (TMAO), **(E)** homocysteine (Hcy), and **(F)** neopterin (Neo). Data represent the mean and SD, with individual data points identified as circles (standard diet) and triangles (resistant protein diet). Statistical difference between groups is designated as follows: *p < 0.05.

Compared with the standard diet group, metabolites analysis identified that tyrosine, homocysteine, tri-methylamine-N-oxide (TMAO), and neopterin were significantly increased, and acetic acid was significantly lowered in the resistant protein diet group (Figure 7, and Supplementary Table 4). Several additional biomarkers trended towards differentiation (Supplementary Figure 1) but statistical significance was not reached likely due to the limited (*n* = 3) replicates analysed for each treatment.

## Discussion

**Figure 8:**
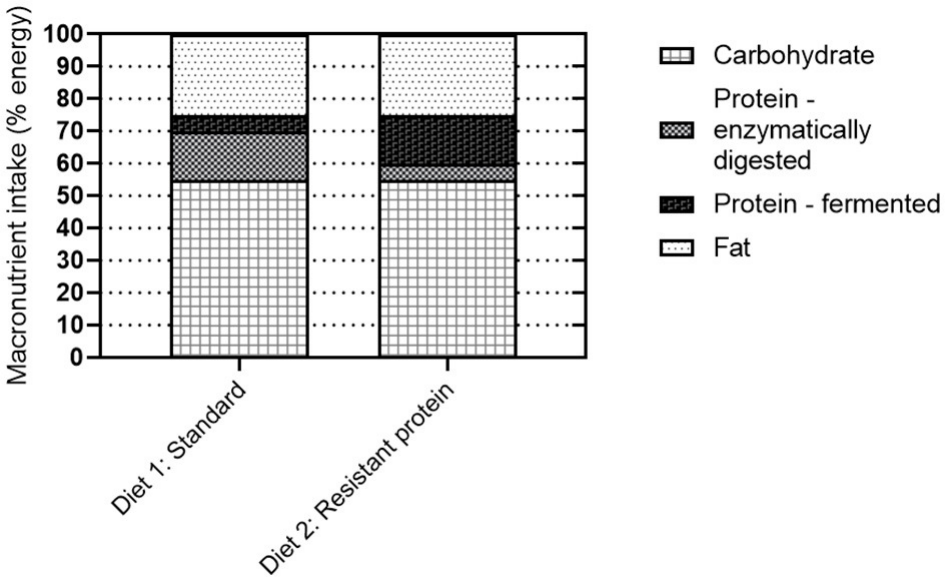
Schematic demonstrating the proportion of protein that is enzymatically digested vs. fermented by the gut microbiota as a function of protein resistance (standard or resistant protein).

This study aimed to determine the effects of substituting a standard form of dietary protein with a resistant form of protein, produced by the high-heat treatment of the feed. In both diets, the total protein was 21% of total solids, as required for the developmental stage of life of the pigs. As such, the study did not compare the effects of a high with standard protein diet, but modelled the acute response to colonic fermentation of indigestible, resistant protein (Figure 8), which is present in many processed protein-containing foods, albeit in lower proportions. Here we begin to address the missing links between a high resistant protein diet, the fermentation of resistant proteins, and adverse health outcomes.

### Digestibility and Bioavailability of Resistant Protein

Standard food processing techniques, such as heating, are known to compromise enzymatic digestibility and promote protein digestive resistance. For example, the prolonged heating of pre-cooked tuna (160–180°C for up to 3 h) caused significant time and temperature-dependent reductions in the enzymatic digestibility of protein (44). Similarly, the prolonged heating of a model protein, albumin, with formaldehyde or glucose caused significantly lowered protein digestibility, as demonstrated in rats and chicks (45). In the current study, the lowering of protein digestibility, i.e., digestive resistance, caused by heating was demonstrated by the loss of *in vitro* enzymatic digestibility (Figure 2A).

The high-heat processing of protein-containing foods results in reduced protein digestibility (resistant proteins) and the chemical products of Maillard reactions between proteins and carbohydrates, known as Maillard reaction products (MRPs) (8, 46). In a cohort of 20 adolescent boys fed a diet either low or high in MRPs for 2 weeks, 47% higher faecal nitrogen and 12% lower apparent nitrogen absorption was observed following the high-MRP diet (47). This indicates that the protein (nitrogen) from the high-MRP processed diet was resistant to enzymatic digestion and reached the colon where it could be fermented by the microbiota, and later excreted. While the excessive consumption of dietary MRPs (particularly advanced glycation end-products) is known to lead to an inflammatory phenotype (48), the effects of resistant protein fermentation on colonic or host health are less defined.

The concept of resistant protein that is incompletely digested in the small intestine and fermented in the colon, has been presented previously. While early reports suggested that protein fermentation was beneficial (5), the emerging consensus is that the fermentation of resistant protein is unfavourable for the reasons of (i) forfeited protein nutrition and (ii) fermentation metabolites, such as ammonia, phenols, indoles, sulphides, and biogenic amines, which are generally considered harmful (8, 10, 21–23). The current study confirmed that feeding resistant protein to young pigs had an adverse effect on body weight gain (Figure 2B), which may be partly due to reduced feed intake (Figure 2C). This demonstrates the forfeited protein nutrition that results from a resistant protein diet (Figure 8, diet 2) compared with a standard protein diet (Figure 8, diet 1). The following sections discuss the consequences of protein fermentation and the resulting metabolites on host health.

### Effects of Resistant Protein on the Gut Microbiome

The focus of this research was to investigate the effects of resistant protein fermentation on gut microbiota composition, the resulting metabolites, and host health. To avoid the confounding effect of altering the ratio of dietary protein to carbohydrates, which can in itself affect the microbiota, energy content and macronutrient ratios were kept consistent between the diets. Therefore, shifts in microbiome and their metabolites were specific to altering the proportion of resistant to digestible protein (Figure 8). The colonic fermentation of protein is known to influence microbial biodiversity in the favour of species that catabolise proteins, peptides, and amino acids. In athletes, the use of a protein supplement (containing processed proteins) altered the composition of the microbiome, increasing the abundance of the *Bacteroidetes* phylum (49). Similarly, in the present study, the abundance of *Bacteroidetes* was higher (though not significantly) in the resistant protein diet group compared with the standard diet group (Figure 6).

However, more important than differences in certain phylum, are the differences in individual taxa. The capacity for pig gut microbiota to utilise protein has been previously demonstrated (50), and a correlation between total faecal bacteria (particularly faecal *Lactobacillus*) and nitrogen utilisation efficiency has been observed (50). Similarly, an elevation of the *Lactobacillus* taxa was observed in the resistant protein diet group in the present study (Supplementary Figure 2), suggesting an increased utilisation of nitrogen. However, the increased levels of *Lactobacillus* may also have been due to the milk powder in the resistant protein diet feed, which was absent from the standard diet.

The present study indicated a high prevalence of *Prevotella* genus among pigs on the resistant protein diet. A *Prevotella-*favouring enterotype has been associated with a low animal protein and saturated fat diet, and high carbohydrate and simple sugars diet. The *Prevotella* enterotype was associated with being vegetarian (51). While this initially appears to be in contrast with the present findings, it may actually support the idea that when dietary protein is more resistant to digestion (i.e., plant-based protein compared with the animal sources of protein), this encourages an expansion of the *Prevotella* genus. Similarly, data from Dong et al. (52) supports the conclusion that the increased microbial utilisation of protein is associated with an increase in *Prevotella*. They observed that faecal *Prevotella_7 spp* was depleted in people with adequate fibre intake and increased in people with higher protein intake (52). However, contrasting findings from a study on the effects of a high protein diet on the microbiota of rats, showed a reduction in *Prevotella* among the rats on the high protein diet, compared with the normal protein diet (53).

Differences in β-diversity were observed between the diet groups in the present study (Figure 5), indicating the dissimilarity in microbial communities caused by the increased levels of indigestible protein reaching the colon in the resistant protein diet compared with the standard diet. Previously, shifts in β-diversity have been shown in response to high protein diets (16), supporting that bacterial communities readily adjust to utilise the substrate that is available to them. For example, compared with a standard protein diet fed to healthy mice (19.4% energy), a high protein diet (52% energy) altered several measures of β-diversity, without affecting α-diversity (16). Additionally, predicted metagenome analysis demonstrated increases in the urea cycle pathway with the high protein diet, providing an indication of increased nitrogen utilisation by the microbial community in response to the increase in dietary protein (16).

### Effects of Resistant Protein on Plasma Metabolites

The microbial fermentation of protein is known to produce various compounds, such as biogenic amines, short- and branched-chain fatty acids, ammonia, phenols, cresols, indoles, and sulphides (10, 20–23). A problem with high intake of resistant protein is that many products of protein fermentation are toxic at increased levels (7). Here, we report plasma markers that reflect protein metabolites produced by the microbiota and absorbed into the bloodstream.

#### Biogenic Amines

It was expected that the fermentation of resistant protein by the gut microbiota would produce biogenic amines, as this has been previously demonstrated within the intestinal environment. Luo et al. (54) observed that, in comparison with a normal protein diet (20%), a low protein diet (14%) fed to piglets for 45 days reduced the levels of cadaverine (a biogenic amine) in the caecal contents. This suggests that the production of biogenic amines by the gut microbiota is proportional to the amount of protein available for fermentation. Similarly, a study in which piglets were fed diets consisting of 17, 19, or 23% protein, reported increased biogenic amines; putrescine, histamine, and spermidine, in the colonic contents of piglets on the 23% protein diet, compared with 17 and 19% protein (55). Furthermore, the level of tryptamine in the caecal contents of broiler chickens was significantly increased following a high resistant protein diet, compared with a low resistant protein diet, however, the level of total amines was lower following the high resistant protein diet (56). In the current study, tyramine trended towards elevation in plasma in the resistant protein diet group (Supplementary Figure 1A). Collectively, these results indicate that the fermentation of resistant protein in the colon results in the production of biogenic amines, which can be absorbed and enter the host's bloodstream.

#### Short-Chain Fatty Acids

The fermentation of resistant protein in the large intestine produces a range of short- and branched-chain fatty acids (57). In the present study, the plasma levels of all measured short-chain fatty acids trended to be lower, while acetic acid was significantly lower (*p* < 0.05), following the resistant protein diet compared with the standard diet (Supplementary Figure 1C). A similar effect was observed by Bryan et al. (56) in broiler chickens fed with high- or low-resistant protein diets, where the level of short-chain fatty acids was reduced following the high-resistant protein diet. The lower levels of acetic acid, a beneficial short-chain fatty acid, and one of the dominant short chain fatty acids produced by the fermentation of fibre in the gut (58), likely reflected the altered microbiota profile and altered fermentation substrate with the resistant protein diet. This was observed despite similar levels of fibre in the standard and resistant protein diets. Short-chain fatty acids, as well as providing energy to intestinal cells, play a role in preventing pathogenic bacterial growth, maintaining intestinal barrier function, supporting intestinal immune function, regulating fat and cholesterol synthesis in the liver, and suppressing weight gain by promoting glucagon secretion (58). The lowered levels of short-chain fatty acids observed following the resistant protein diet may, therefore, be associated with the poorer health status and increased risk of disease.

#### Uremic Solutes

The key indicators of health impact from protein-modified diets are the levels of amino acid-derived metabolites, which are associated with toxicity (7). The present findings indicated that plasma p-cresol, p-cresol sulphate, p-cresol glucuronide, and indoxyl sulphate were increased in the resistant protein diet group, although, likely due to the small sample size (*n* = 3), were not increased statistically (Supplementary Figure 1D). This is in line with a previous research, which demonstrated the production of indole and p-cresol compounds following the fermentation of proteins and peptones using faecal microbiota from healthy humans, in a model of the fermentative catabolism of intact protein (59). Indoxyl sulphate, a microbial metabolite of tryptophan (sulphated in the liver), is associated with accelerated glomerular sclerosis, endothelial dysfunction, enhanced monocyte adhesion to the vascular endothelium, and increased oxidative stress (60). Indoxyl sulphate and p-cresol sulphate (a microbial metabolite of tyrosine) both act as uremic toxins, with cytotoxic activity towards renal cells (11). In the kidney, p-cresol sulphate induces pro-inflammatory changes in cytokine mRNA expression and promotes proximal tubular cell death (61). The increased levels of p-cresol sulphate are associated with poorer clinical outcomes for patients with chronic kidney disease and were correlated with cardiovascular mortality (60). These findings indicate that resistant protein fermentation produces potentially toxic compounds, such as indoles and cresols that can enter the host's bloodstream.

### Effects of Resistant Protein on Health and Disease Risk

The adverse effects of excessive colonic protein fermentation may be attributed to the development of a pathogenic and pro-inflammatory microbial phenotype that lowers short-chain fatty acid production and increases the levels of amines, phenols, sulphides, indoles, and other bioactive derivatives of aromatic amino acids (7, 10). The following section describes the effect of the resistant protein diet on health and disease risk as indicated by the markers of systemic responses by the host.

#### Chronic Kidney Disease

Tyrosine, homocysteine, tri-methylamine-N-oxide, a uremic retention solute that is associated with renal dysfunction, inflammation, oxidative stress, and mortality in chronic kidney disease (CKD) (62), was increased in the resistant protein diet group (Figure 7). TMAO is formed through the microbial metabolism of carnitine (and choline) into trimethylamine, which is converted in the liver to TMAO (62). Similarly, neopterin, a marker of cell-mediated immunity, is also associated with renal dysfunction, inflammation, oxidative stress, and mortality in CKD (62), and was increased in the resistant protein diet group (Figure 7). A strong elevation of neopterin suggests a cellular immune response consistent with CKD in the pigs (63, 64). The metabolomics profile exhibited by pigs in the resistant protein diet group strongly indicated the pathology of CKD. The resistant protein diet group had worsened renal function as evidenced by lower eGFR (Figure 3).

#### Inflammation

Compared with the standard diet, several markers of pro-inflammatory status (homocysteine and neopterin) were increased in the resistant protein diet group (Figure 7). Chronic exposure to dietary MRPs is understood to contribute to multiple inflammation-driven degenerative diseases, *via* antagonism of the advanced glycation end-product receptor (65) and, more recently, due to the allergenicity of MRPs (66). In a similar study, rats fed with a high-MRP skim milk powder exhibited the increased levels of mediators associated with intestinal inflammation (67). In further support, an extreme high protein diet (52% of energy) fed to mice, produced the elevation of multiple plasma biomarkers of inflammation (nuclear factor-kappa B, monocyte chemoattractant protein-1, and tumour necrosis factor-α) and adversely affected both intestinal permeability and kidney function (16). Collectively, this data suggest that chronic exposure to excess process-modified protein (e.g., MRPs and resistant protein), and the microbial fermentation of that protein, contributes to chronic inflammation.

### Limitations

This study demonstrated the novel effects of a diet high in resistant protein. While the design of the feeds and range of outcome markers measured were a strength of the study, we acknowledge several limitations. The sample size was small with 4 pigs per group and this was further reduced for certain analyses where only 3 pigs from each group were included due to the sample collection process or limitations in analysis capacity. The lower feed intake, due to an apparent and unforeseen dislike for the feed, in the resistant protein diet group was likely responsible for the reduced growth and weight gain, meaning this cannot be attributed to the poor bioavailability of the resistant protein. The lack of feed intake in this group may have indirectly affected other markers of metabolism and health status. Furthermore, the thiamine deficiency that resulted from the nutrient loss caused by the heating of resistant protein feed may also have negatively influenced the health status and feed intake of the pigs in the resistant protein diet group (68). Further investigation of the key findings from this work is required to gain an understanding of the health impacts of a diet high in resistant protein and the mechanisms by which they occur.

## Conclusion

This study has demonstrated the effects of feeding a resistant protein diet, that modelled a highly processed and non-bioavailable form of protein, to pigs for 4 weeks on gut microbial composition, microbial metabolites, and markers of health status. The resistant protein was approximately half as digestible as the standard protein, with the lower bioavailability, reflected by the significantly lower gain of body mass. The consumption of resistant protein diet induced a shift in the composition of the gut microbiome and the elevation of protein-derived fermentation metabolites, confirming that the fermentation of resistant protein selects for a distinct microbial assemblage and promotes the production of nitrogenous metabolites. Plasma metabolomic analysis indicated that the levels of tyrosine, TMAO, homocysteine, and neopterin were increased, and the levels of acetic acid were reduced in the resistant protein diet group. In addition, responses to the resistant protein diet indicated reduced renal function and increased risk of CKD. In summary, the resistant protein diet appeared to invoke gut microbiome and host-mediated responses that contributed to risk factors for chronic diseases. Although the diet fed to pigs in the current study represented an extreme diet containing the high levels of resistant protein, there is some level of resistant protein present in most protein-containing processed foods and it is expected that habitual consumption over a longer period could drive the biological processes observed under these research conditions. The results justify the need to increase awareness and monitor the levels of resistant protein in processed foods and their potential relationship with adverse health outcomes.

## Data Availability Statement

The original contributions presented in the study are publicly available. This data can be found here: https://www.ncbi.nlm.nih.gov/bioproject/798448.

## Ethics Statement

The animal study was reviewed and approved by Monash Animal Research Platform-1 Animal Ethics Committee Monash University, Clayton 3800, Australia.

## Author Contributions

MM, SS-P, and LB: conceptualization. MM, AG, SS-P, GW, and LB: methodology. MM and VK: formal analysis. MM, AG, VK, MS, KT, TMW, TW, and LB: investigation. MC, FZM, and LB: resources. MM, MS, and KT: data curation. MM and LB: writing—original draft. MM, MC, AG, VK, FZM, SS-P, MS, KT, GW, TW, and LB: writing—review and editing. MM, MS, KT, TW, and LB: visualization. MC, FZM, GW, TMW, and LB: supervision. MM: project administration. MC, FZM, SS-P, TMW, and LB: funding acquisition. All authors have read and agreed to the published version of the manuscript.

## Funding

This project was funded by Monash University under the Fraunhofer-Gesellschaft's ICON program. FZM was supported by a grant of the National Health & Medical Research Council (NHMRC) (1159721), National Heart Foundation Future Leader Fellowship (101185) and Vanguard Grants, and by a Senior Medical Research Fellowship from the Sylvia and Charles Viertel Charitable Foundation Fellowship. MC is the recipient of a Career Development Award from JDRF Australia (4-CDA-2018-613-M-B), the recipient of the Australian Research Council Special Research Initiative in Type 1 Juvenile Diabetes.

## Conflict of Interest

The authors declare that the research was conducted in the absence of any commercial or financial relationships that could be construed as a potential conflict of interest.

## Publisher's Note

All claims expressed in this article are solely those of the authors and do not necessarily represent those of their affiliated organizations, or those of the publisher, the editors and the reviewers. Any product that may be evaluated in this article, or claim that may be made by its manufacturer, is not guaranteed or endorsed by the publisher.
