# Supplementary material for: Reduced Growth, Altered Gut Microbiome and Metabolite Profile, and Increased Chronic Kidney Disease Risk in Young Pigs Consuming a Diet Containing Highly Resistant Protein
Source: Front Nutr. 2022 Mar 24;9:816749. doi: 10.3389/fnut.2022.816749 (PMC8988180; doi:10.3389/fnut.2022.816749)
Supplement: Supplementary file 1 [file Data_Sheet_1.docx]

Supplementary Material

**Supplementary Table 1.** Compositional profiles of standard and resistant protein pig weaner diets.

|  | **Standard diet** | **Resistant protein diet** |
| --- | --- | --- |
| Ingredient summary | Wheat, lupins, soya meal, barley, calcium carbonate, salt, dicalcium phosphate, lysine, and a vitamin and trace mineral premix | Skim milk, barley, canola meal, soya meal, calcium carbonate, salt, dicalcium phosphate, and a vitamin and trace mineral premix |
| Autoclaved | No | Yes |
| **Nutritional parameters** |  |  |
| Digestible energy (kJ/g) | 13.3 | 13.3 |
| Protein (%) | 21 | 21 |
| Total fat (%) | 2.3 | 2.2 |
| Crude fibre (%) | 3.9 | 3.6 |
| Acid detergent fibre (%) | 6.5 | 10 |
| Calcium (%) | 0.7 | 0.8 |
| Phosphate (%) | 0.6 | 0.7 |
| **Added trace minerals as fed** |  |  |
| Magnesium (mg/kg) | 230 | 230 |
| Iron (mg/kg) | 140 | 142 |
| Copper (mg/kg) | 30 | 30 |
| Iodine (mg/kg) | 1.1 | 1 |
| Manganese (mg/kg) | 150 | 150 |
| Cobalt (mg/kg) | 0.3 | 0.3 |
| Zinc (mg/kg) | 160 | 160 |
| Molybdenum (mg/kg) | 0.7 | 0.7 |
| Selenium (mg/kg) | 0.7 | 0.6 |
| **Added vitamins as fed** |  |  |
| Vitamin A (retinol) (iu/kg) | 17,900 | 17,900 |
| Vitamin D (cholecalciferol) (iu/kg) | 3,590 | 3,590 |
| Vitamin E (a tocopherol acetate) (mg/kg) | 160 | 160 |
| Vitamin K (menadione) (mg/kg) | 30 | 30 |
| Vitamin B1 (thiamine) (mg/kg) | 120 | 120 |
| Vitamin B2 (riboflavin) (mg/kg) | 47 | 47 |
| Niacin (nicotinic acid) (mg/kg) | 155 | 154 |
| Vitamin B6 (pryridoxine) (mg/kg) | 38 | 38 |
| Calcium pantothenate (mg/kg) | 78 | 78 |
| Biotin (ug/kg) | 455 | 455 |
| Folic acid (mg/kg) | 8 | 8 |
| Vitamin B12 (cyanocobalamin) (ug/kg) | 230 | 230 |
| Choline (mg/kg) | 44 | 45 |
| **Calculated essential amino acids as fed** |  |  |
| Valine (%) | 0.93 | 1.11 |
| Leucine (%) | 1.44 | 1.63 |
| Isoleucine (%) | 0.80 | 0.88 |
| Threonine (%) | 0.72 | 0.8 |
| Methionine (%) | 0.28 | 0.36 |
| Cysteine (%) | 0.34 | 0.27 |
| Lysine (%) | 1.00 | 1.18 |
| Phenylalanine (%) | 0.96 | 0.98 |
| Tyrosine (%) | 0.68 | 0.81 |
| Tryptophan (%) | 0.21 | 0.23 |
| Histidine (%) | 0.50 | 0.55 |
| **Calculated total minerals as fed** |  |  |
| Calcium (%) | 0.7 | 0.8 |
| Phosphorous (%) | 0.64 | 0.7 |
| Available phosphorous (%) | 0.3 | 0.48 |
| Magnesium (%) | 0.25 | 0.22 |
| Sodium (%) | 0.17 | 0.31 |
| Chloride (%) | 0.34 | 0.4 |
| Potassium (%) | 1.02 | 1.04 |
| Sulphur (%) | 0.2 | 0.14 |
| Iron (mg/kg) | 242 | 210 |
| Copper (mg/kg) | 40 | 38 |
| Iodine (mg/kg) | 1.1 | 1.1 |
| Manganese (mg/kg) | 190 | 172 |
| Cobalt (mg/kg) | 0.6 | 0.3 |
| Zinc (mg/kg) | 200 | 200 |
| Selenium (mg/kg) | 0.9 | 0.8 |
| Molybdenum (mg/kg) | 0.7 | 0.7 |
| Cadmium (mg/kg) | Trace | Trace |
| Chromium (mg/kg) | No data | No data |
| Boron (mg/kg) | No data | No data |
| **Calculated total vitamins as fed** |  |  |
| Vitamin A (retinol) (iu/kg) | 18,000 | 18,000 |
| Vitamin D (cholecalciferol) (iu/kg) | 3,580 | 3,590 |
| Vitamin E (a tocopherol acetate) (mg/kg) | 178 | 185 |
| Vitamin K (menadione) (mg/kg) | 30 | 30 |
| Vitamin C (ascorbic acid) | No data | No data |
| Vitamin B1 (thiamine) (mg/kg) | 125 | 124 |
| Vitamin B2 (riboflavin) (mg/kg) | 49 | 53 |
| Niacin (nicotinic acid) (mg/kg) | 210 | 200 |
| Vitamin B6 (pyridoxine) (mg/kg) | 40 | 40 |
| Pantothenic acid (mg/kg) | 87 | 90 |
| Biotin (ug/kg) | 680 | 650 |
| Folic acid (mg/kg) | 9 | 9 |
| Vitamin B12 (cyanocobalamin) (ug/kg) | 228 | 230 |
| Choline (mg/kg) | 1660 | 1,430 |
| **Calculated fatty acid composition as fed** |  |  |
| Palmitic acid 16:0 (%) | 0.29 | 0.39 |
| Stearic acid 18:0 (%) | 0.01 | 0.04 |
| Palmitoleic acid 16:1 (%) | Trace | Trace |
| Oleic acid 18:1 (%) | 0.45 | 0.52 |
| Gadoleic acid 20:1 (%) | Trace | Trace |
| Linoleic Acid 18:2 n6 (%) | 0.76 | 0.75 |
| Α Linolenic Acid 18:3 n3 (%) | 0.12 | 0.11 |
| Arachidonic Acid 20:4 n6 (%) | No data | No data |
| EPA 20:5 n3 (%) | No data | No data |
| DHA 22:6 n3 (%) | No data | No data |
| Total n3 (%) | 0.12 | 0.11 |
| Total n6 (%) | 0.76 | 0.75 |
| Total mono unsaturated fats (%) | 0.46 | 0.54 |
| Total polyunsaturated fats (%) | 0.88 | 0.86 |
| Total saturated fats (%) | 0.3 | 0.49 |

DHA, docosahexaenoic acid; EPA, eicosapentaenoic acid

**Supplementary Table 2.** Summary of blood biomarker analyses for standard diet group and resistant protein diet group taken at the end of the study period.

|  |  | **Standard diet** | | | **Resistant protein diet** | | | Difference  (*p* value) |
| --- | --- | --- | --- | --- | --- | --- | --- | --- |
|  | Unit | N | Mean | SD | N | Mean | SD |  |
| **GENERAL HAEMATOLOGY** | |  |  |  |  |  |  |  |
| **Red cell count** | x 10^12^/L | 3 | 5.08 | 0.38 | 2 | 4.00 | 0.33 | **0.047** |
| Haemoglobin | g/L | 3 | 93.00 | 6.08 | 2 | 72.50 | 4.95 | **0.030** |
| Hct | L/L | 3 | 0.28 | 0.02 | 2 | 0.22 | 0.01 | **0.028** |
| MCV | fL | 3 | 54.67 | 1.15 | 2 | 54.00 | 1.41 | 0.599 |
| MCH | pg | 3 | 18.00 | 0.00 | 2 | 18.00 | 0.00 | NA |
| MCHC | g/L | 3 | 334.00 | 5.20 | 2 | 337.00 | 0.00 | 0.495 |
| Reticulocytes | x 10^9^/L | 3 | 296.33 | 82.31 | 2 | 298.00 | 21.21 | 0.980 |
| **White cell count** | x 10^9^/L | 3 | 16.03 | 3.48 | 2 | 18.00 | 0.28 | 0.504 |
| Neutrophils | x 10^9^/L | 3 | 7.83 | 4.02 | 2 | 12.10 | 2.97 | 0.296 |
| Band forms | x 10^9^/L | 3 | 0.00 | 0.00 | 2 | 0.00 | 0.00 | NA |
| Lymphocytes | x 10^9^/L | 3 | 6.53 | 5.56 | 2 | 4.90 | 3.11 | 0.738 |
| Monocytes | x 10^9^/L | 3 | 1.30 | 1.01 | 2 | 0.85 | 0.64 | 0.624 |
| Eosinophils | x 10^9^/L | 3 | 0.40 | 0.30 | 2 | 0.10 | 0.14 | 0.293 |
| Basophils | x 10^9^/L | 3 | 0.00 | 0.00 | 2 | 0.10 | 0.14 | 0.272 |
| **Platelets** | x 10^9^/L | 3 | 451.67 | 240.20 | 2 | 463.00 | NA | 0.971 |
| Fibrinogen manual | g/L | 3 | 1.97 | 0.45 | 2 | 2.00 | 0.14 | 0.929 |
|  |  |  |  |  |  |  |  |  |
| **GENERAL BIOCHEMISTRY** | |  |  |  |  |  |  |  |
| Sodium | mmol/L | 3 | 143.67 | 4.04 | 3 | 143.67 | 2.08 | 1.000 |
| Potassium | mmol/L | 3 | 4.77 | 0.74 | 3 | 4.97 | 0.42 | 0.703 |
| Na/K |  | 3 | 30.67 | 4.94 | 3 | 29.07 | 2.91 | 0.654 |
| Chloride | mmol/L | 3 | 100.00 | 2.65 | 3 | 101.00 | 1.00 | 0.573 |
| Bicarbonate | mmol/L | 3 | 36.00 | 3.61 | 3 | 35.33 | 1.53 | 0.783 |
| Anion gap | mmol/L | 3 | 12.67 | 2.89 | 3 | 12.33 | 1.53 | 0.868 |
| Urea | mmol/L | 3 | 4.63 | 1.26 | 3 | 4.87 | 1.79 | 0.862 |
| Creatinine | umol/L | 3 | 59.00 | 2.65 | 3 | 48.00 | 4.36 | **0.020** |
| Calcium | mmol/L | 3 | 2.63 | 0.09 | 3 | 2.60 | 0.00 | 0.645 |
| Phosphate | mmol/L | 3 | 3.43 | 0.58 | 3 | 2.07 | 0.82 | 0.079 |
| Magnesium | mmol/L | 3 | 1.03 | 0.06 | 3 | 1.07 | 0.06 | 0.519 |
| GLDH | U/L | 3 | 2.00 | 0.00 | 3 | 3.00 | 2.83 | 0.653 |
| B-OH Butyrate | mmol/L | 3 | 0.00 | 0.00 | 3 | 0.00 | 0.00 | NA |
| Protein | g/L | 3 | 48.67 | 2.08 | 3 | 40.67 | 1.53 | **0.006** |
| Albumin | g/L | 3 | 33.33 | 0.58 | 3 | 23.33 | 1.53 | **<0.001** |
| Globulin | g/L | 3 | 15.33 | 2.31 | 3 | 17.33 | 0.58 | 0.219 |
| ALP | U/L | 3 | 244.67 | 30.92 | 3 | 219.67 | 2.89 | 0.236 |
| GGT | U/L | 3 | 46.33 | 19.30 | 3 | 55.33 | 26.03 | 0.656 |
| AST | U/L | 3 | 40.00 | 4.36 | 3 | 50.67 | 6.43 | 0.076 |
| CK | U/L | 3 | 396.67 | 20.60 | 3 | 468.67 | 94.71 | 0.268 |
| Cholesterol | mmol/L | 3 | 1.83 | 0.49 | 3 | 2.20 | 0.10 | 0.276 |

Hct, haematocrit; MCV, mean corpuscular volume; MCH, mean corpuscular haemoglobin; MCHC, mean corpuscular haemoglobin content; GLDH, glutamate dehydrogenase; ALP, alkaline phosphatase; GGT, gamma-glutamyl transferase; AST, aspartate aminotransferase; CK, creatinine kinase; NA, not available

**Supplementary Table 3.** Summary of 121 taxa that were significantly different between the standard and resistant diet groups after adjustment for multiple comparisons (*q*<0.05, n=3 per group)

| **Name** | **Base mean** | **Log 2 Fold Change** | **Log Fold Change-SE** | **Stat** | **Adjusted p value** | **Direction of change in resistant protein diet** |
| --- | --- | --- | --- | --- | --- | --- |
| Bacteroidetes;Bacteroidia;Bacteroidales;Prevotellaceae;Prevotella;950818 | 23.2061 | 22.1370 | 3.2375 | 6.8376 | 0.0000 | up |
| Firmicutes;Bacilli;Lactobacillales;Lactobacillaceae;Lactobacillus;5c4f0d | 3423.2790 | -15.3943 | 2.3097 | -6.6651 | 0.0000 | down |
| Firmicutes;Bacilli;Lactobacillales;Streptococcaceae;Streptococcus;f8e25f | 2350.0426 | 14.7157 | 2.2332 | 6.5894 | 0.0000 | up |
| Firmicutes;Bacilli;Lactobacillales;Lactobacillaceae;Lactobacillus;4bfe46 | 332.3435 | -12.0296 | 2.2975 | -5.2359 | 0.0000 | down |
| Firmicutes;Bacilli;Lactobacillales;Lactobacillaceae;Lactobacillus;d9702a | 355.9325 | 11.0302 | 2.1199 | 5.2032 | 0.0000 | up |
| Firmicutes;Clostridia;Clostridiales;Clostridiaceae_1;Clostridium_sensu_stricto;2f56e1 | 201.0945 | -11.3047 | 2.2442 | -5.0373 | 0.0001 | down |
| Bacteroidetes;Bacteroidia;Bacteroidales;Prevotellaceae;Prevotella;7151bf | 220.4035 | 9.1780 | 2.0437 | 4.4909 | 0.0005 | up |
| Firmicutes;Bacilli;Lactobacillales;Lactobacillaceae;Lactobacillus;1db5e5 | 155.3798 | -10.9330 | 2.4224 | -4.5133 | 0.0005 | down |
| Firmicutes;Bacilli;Lactobacillales;Lactobacillaceae;Lactobacillus;60e8e0 | 152.3165 | 8.7646 | 1.9594 | 4.4732 | 0.0005 | up |
| Firmicutes;Bacilli;Lactobacillales;Lactobacillaceae;Lactobacillus;cfcdc8 | 89.2896 | 9.9969 | 2.2394 | 4.4640 | 0.0005 | up |
| Firmicutes;Clostridia;Clostridiales;Lachnospiraceae;NA;2b4569 | 277.1874 | 9.6694 | 2.1745 | 4.4467 | 0.0005 | up |
| Firmicutes;Clostridia;Clostridiales;Peptostreptococcaceae;Romboutsia;98f93a | 87.5728 | -10.1052 | 2.3244 | -4.3474 | 0.0008 | down |
| Firmicutes;Bacilli;Lactobacillales;Lactobacillaceae;Lactobacillus;3675ec | 82.9594 | -10.0273 | 2.3377 | -4.2895 | 0.0009 | down |
| Firmicutes;Erysipelotrichia;Erysipelotrichales;Erysipelotrichaceae;Kandleria;e8e9c1 | 2605.8243 | 9.4492 | 2.2192 | 4.2578 | 0.0010 | up |
| Firmicutes;Clostridia;Clostridiales;Ruminococcaceae;NA;24e637 | 52.6247 | -9.3712 | 2.2355 | -4.1919 | 0.0012 | down |
| Firmicutes;Bacilli;Lactobacillales;Streptococcaceae;Streptococcus;6d82a6 | 59.6212 | 9.4152 | 2.2591 | 4.1676 | 0.0013 | up |
| Firmicutes;Bacilli;Lactobacillales;Streptococcaceae;Streptococcus;5851e9 | 54.0521 | 9.2731 | 2.2563 | 4.1098 | 0.0016 | up |
| Firmicutes;Clostridia;Clostridiales;Clostridiales_Incertae_Sedis_XIII;NA;df4e3a | 55.2091 | -9.4408 | 2.3341 | -4.0447 | 0.0017 | down |
| Firmicutes;Clostridia;Clostridiales;Lachnospiraceae;Pseudobutyrivibrio;28ab1b | 250.3487 | 11.4847 | 2.8468 | 4.0342 | 0.0017 | up |
| Firmicutes;Clostridia;Clostridiales;Peptostreptococcaceae;Terrisporobacter;701764 | 67.3063 | -8.7243 | 2.1451 | -4.0671 | 0.0017 | down |
| Firmicutes;Erysipelotrichia;Erysipelotrichales;Erysipelotrichaceae;Faecalitalea;53895e | 92.9137 | 9.0893 | 2.2509 | 4.0380 | 0.0017 | up |
| Spirochaetes;Spirochaetia;Spirochaetales;Spirochaetaceae;Treponema;e05cb5 | 43.5206 | -9.0967 | 2.2558 | -4.0326 | 0.0017 | down |
| Firmicutes;Bacilli;Lactobacillales;Streptococcaceae;Streptococcus;9e7bba | 47.3761 | 9.0842 | 2.2771 | 3.9893 | 0.0019 | up |
| Firmicutes;Erysipelotrichia;Erysipelotrichales;Erysipelotrichaceae;Faecalicoccus;6fa390 | 39.2012 | -8.9454 | 2.2610 | -3.9564 | 0.0021 | down |
| Firmicutes;Clostridia;Clostridiales;Eubacteriaceae;Eubacterium;7cff73 | 69.5307 | 7.8062 | 2.0021 | 3.8990 | 0.0026 | up |
| Firmicutes;Clostridia;Clostridiales;Lachnospiraceae;Blautia;36f7d6 | 143.1280 | 10.6779 | 2.8209 | 3.7852 | 0.0040 | up |
| Firmicutes;Bacilli;Lactobacillales;Lactobacillaceae;Lactobacillus;0dd608 | 28.7061 | -8.4960 | 2.2567 | -3.7647 | 0.0041 | down |
| Bacteroidetes;Bacteroidia;Bacteroidales;Prevotellaceae;Prevotella;44d47b | 80.1232 | 7.8772 | 2.0987 | 3.7534 | 0.0042 | up |
| Actinobacteria;Actinobacteria;Bifidobacteriales;Bifidobacteriaceae;Bifidobacterium;63367d | 35.7320 | -8.8112 | 2.3546 | -3.7421 | 0.0042 | down |
| Bacteroidetes;Bacteroidia;Bacteroidales;Prevotellaceae;Prevotella;4888db | 150.6861 | 7.4339 | 2.0727 | 3.5865 | 0.0063 | up |
| Firmicutes;Clostridia;Clostridiales;Lachnospiraceae;NA;4955b4 | 304.5275 | 9.6585 | 2.6812 | 3.6023 | 0.0063 | up |
| Firmicutes;Clostridia;Clostridiales;Ruminococcaceae;Ruminococcus;7bfb4d | 24.1875 | 8.1142 | 2.2600 | 3.5904 | 0.0063 | up |
| Firmicutes;Clostridia;Clostridiales;Ruminococcaceae;Ruminococcus;9d3807 | 48.4977 | 9.1156 | 2.5438 | 3.5834 | 0.0063 | up |
| Firmicutes;Negativicutes;Selenomonadales;Veillonellaceae;Mitsuokella;97ea10 | 46.5823 | 8.0908 | 2.2368 | 3.6172 | 0.0063 | up |
| NA;NA;NA;NA;NA;01bde9 | 24.4116 | 8.1273 | 2.2492 | 3.6134 | 0.0063 | up |
| Spirochaetes;Spirochaetia;Spirochaetales;Spirochaetaceae;Treponema;e8f663 | 84.6206 | -10.0564 | 2.7741 | -3.6251 | 0.0063 | down |
| Firmicutes;Clostridia;Clostridiales;Lachnospiraceae;NA;d32268 | 94.2748 | 10.0751 | 2.8310 | 3.5588 | 0.0068 | up |
| Firmicutes;Clostridia;Clostridiales;Lachnospiraceae;Dorea;4277e6 | 91.3707 | -6.2039 | 1.7594 | -3.5261 | 0.0074 | down |
| Firmicutes;Clostridia;Clostridiales;Lachnospiraceae;Ruminococcus2;b39e0d | 35.3232 | -7.7897 | 2.2277 | -3.4967 | 0.0081 | down |
| Firmicutes;Erysipelotrichia;Erysipelotrichales;Erysipelotrichaceae;Bulleidia;12d17f | 73.2590 | 9.7118 | 2.7871 | 3.4846 | 0.0083 | up |
| Firmicutes;Clostridia;Clostridiales;Clostridiaceae_1;Clostridium_sensu_stricto;7ca75b | 21.4180 | -8.0727 | 2.3452 | -3.4423 | 0.0094 | down |
| Firmicutes;Clostridia;Clostridiales;Lachnospiraceae;Blautia;6e76ab | 28.7293 | 7.3912 | 2.1828 | 3.3861 | 0.0113 | up |
| Firmicutes;Clostridia;Clostridiales;Lachnospiraceae;NA;b66b1d | 98.4810 | 6.2073 | 1.8592 | 3.3387 | 0.0131 | up |
| Bacteroidetes;Bacteroidia;Bacteroidales;Prevotellaceae;Prevotella;20e6e9 | 78.2514 | 5.9869 | 1.8040 | 3.3187 | 0.0138 | up |
| Bacteroidetes;Bacteroidia;Bacteroidales;Prevotellaceae;Prevotella;37965f | 68.7411 | 7.5274 | 2.2934 | 3.2823 | 0.0148 | up |
| Firmicutes;Clostridia;Clostridiales;Lachnospiraceae;NA;21191a | 26.2490 | 8.2326 | 2.5142 | 3.2745 | 0.0148 | up |
| Firmicutes;Clostridia;Clostridiales;NA;NA;6efe51 | 67.9258 | -7.0514 | 2.1519 | -3.2768 | 0.0148 | down |
| Firmicutes;Erysipelotrichia;Erysipelotrichales;Erysipelotrichaceae;Turicibacter;552ecc | 21.0377 | -7.1036 | 2.1587 | -3.2907 | 0.0148 | down |
| Bacteroidetes;Bacteroidia;Bacteroidales;Porphyromonadaceae;Barnesiella;d9775e | 20.3810 | -8.0037 | 2.4658 | -3.2459 | 0.0151 | down |
| Firmicutes;Clostridia;Clostridiales;Ruminococcaceae;Clostridium_IV;94d5cb | 55.2609 | 6.7334 | 2.0694 | 3.2538 | 0.0151 | up |
| Firmicutes;Erysipelotrichia;Erysipelotrichales;Erysipelotrichaceae;Bulleidia;3cae33 | 51.4617 | 9.2008 | 2.8312 | 3.2498 | 0.0151 | up |
| Firmicutes;NA;NA;NA;NA;052e1c | 22.6353 | -7.2105 | 2.2147 | -3.2557 | 0.0151 | down |
| Bacteroidetes;Bacteroidia;Bacteroidales;Porphyromonadaceae;Tannerella;bf27de | 53.1018 | -6.4884 | 2.0060 | -3.2346 | 0.0154 | down |
| Firmicutes;Erysipelotrichia;Erysipelotrichales;Erysipelotrichaceae;Holdemania;a27f02 | 43.2428 | 8.9500 | 2.7770 | 3.2229 | 0.0158 | up |
| Firmicutes;Clostridia;Clostridiales;Lachnospiraceae;NA;00d84b | 14.0148 | 7.3282 | 2.2827 | 3.2104 | 0.0159 | up |
| Firmicutes;Clostridia;Clostridiales;Lachnospiraceae;NA;855a6b | 43.2070 | 8.9486 | 2.7832 | 3.2153 | 0.0159 | up |
| Firmicutes;Clostridia;Clostridiales;Lachnospiraceae;Clostridium_XlVa;ae5f9e | 21.8529 | 6.9947 | 2.2054 | 3.1717 | 0.0178 | up |
| Firmicutes;Negativicutes;Selenomonadales;Veillonellaceae;Mitsuokella;539c46 | 54.0919 | 6.1755 | 1.9509 | 3.1654 | 0.0179 | up |
| Firmicutes;Clostridia;Clostridiales;Ruminococcaceae;NA;58492e | 12.3249 | -7.2787 | 2.3064 | -3.1558 | 0.0182 | down |
| Firmicutes;Clostridia;Clostridiales;Ruminococcaceae;NA;e644aa | 13.1099 | -7.3688 | 2.3473 | -3.1392 | 0.0186 | down |
| Firmicutes;Clostridia;Clostridiales;Ruminococcaceae;NA;fd1cc9 | 16.3963 | -7.6868 | 2.4475 | -3.1407 | 0.0186 | down |
| Bacteroidetes;Bacteroidia;Bacteroidales;Prevotellaceae;Prevotella;9703a9 | 1668.5738 | -5.6129 | 1.8044 | -3.1107 | 0.0196 | down |
| Firmicutes;Clostridia;Clostridiales;Lachnospiraceae;NA;24ffa7 | 48.0980 | 8.1373 | 2.6135 | 3.1136 | 0.0196 | up |
| Firmicutes;Clostridia;Clostridiales;Lachnospiraceae;NA;c2815a | 13.6541 | 7.2814 | 2.3347 | 3.1187 | 0.0196 | up |
| Firmicutes;Negativicutes;Selenomonadales;Veillonellaceae;Dialister;6ad234 | 37.9069 | 8.7589 | 2.8405 | 3.0836 | 0.0211 | up |
| Firmicutes;Erysipelotrichia;Erysipelotrichales;Erysipelotrichaceae;Bulleidia;444b78 | 30.4580 | 8.4435 | 2.7711 | 3.0470 | 0.0235 | up |
| Firmicutes;Clostridia;Clostridiales;Lachnospiraceae;Butyrivibrio;44a28c | 28.9061 | 8.3681 | 2.7660 | 3.0253 | 0.0242 | up |
| Firmicutes;Clostridia;Clostridiales;Lachnospiraceae;NA;c3ef8c | 20.7129 | 6.9195 | 2.2835 | 3.0302 | 0.0242 | up |
| Firmicutes;Clostridia;Clostridiales;Ruminococcaceae;Clostridium_IV;0e2c4c | 10.8700 | -7.0973 | 2.3427 | -3.0296 | 0.0242 | down |
| Bacteroidetes;Bacteroidia;Bacteroidales;Prevotellaceae;Prevotella;4c4a1d | 12.4178 | -7.2866 | 2.4267 | -3.0027 | 0.0253 | down |
| Firmicutes;Clostridia;Clostridiales;NA;NA;7b257b | 12.8940 | 7.2081 | 2.3974 | 3.0067 | 0.0253 | up |
| Bacteroidetes;Bacteroidia;Bacteroidales;Prevotellaceae;Prevotella;b78d0a | 10.6587 | 6.9348 | 2.3255 | 2.9821 | 0.0259 | up |
| Firmicutes;Clostridia;Clostridiales;NA;NA;3626b2 | 11.4565 | 7.0408 | 2.3604 | 2.9828 | 0.0259 | up |
| Firmicutes;Clostridia;Clostridiales;Peptostreptococcaceae;Terrisporobacter;ead5f1 | 9.8667 | -6.9539 | 2.3344 | -2.9789 | 0.0259 | down |
| Firmicutes;Clostridia;Clostridiales;Ruminococcaceae;Intestinimonas;017f2f | 26.1472 | -8.3632 | 2.8081 | -2.9783 | 0.0259 | down |
| Proteobacteria;Deltaproteobacteria;Desulfovibrionales;Desulfovibrionaceae;Desulfovibrio;b33936 | 14.1636 | -6.5334 | 2.1962 | -2.9748 | 0.0259 | down |
| Firmicutes;Clostridia;Clostridiales;Ruminococcaceae;Acetanaerobacterium;9df3cb | 11.3065 | 7.0116 | 2.3680 | 2.9611 | 0.0267 | up |
| Firmicutes;Clostridia;Clostridiales;NA;NA;8f8523 | 12.2478 | -7.2700 | 2.4606 | -2.9546 | 0.0269 | down |
| Firmicutes;Clostridia;Clostridiales;Lachnospiraceae;Butyrivibrio;7e1180 | 28.4286 | 8.3477 | 2.8299 | 2.9498 | 0.0270 | up |
| Euryarchaeota;Methanobacteria;Methanobacteriales;Methanobacteriaceae;Methanobrevibacter;71fb15 | 20.3984 | -7.0617 | 2.4047 | -2.9367 | 0.0278 | down |
| Bacteroidetes;Bacteroidia;Bacteroidales;Prevotellaceae;Prevotella;2dd439 | 45.6729 | -6.2644 | 2.1445 | -2.9211 | 0.0289 | down |
| Firmicutes;Bacilli;Lactobacillales;Lactobacillaceae;Lactobacillus;2e7905 | 8.9096 | -6.8074 | 2.3407 | -2.9083 | 0.0297 | down |
| Firmicutes;Clostridia;Clostridiales;Ruminococcaceae;Ruminococcus;36943b | 25.5339 | 8.1877 | 2.8201 | 2.9034 | 0.0298 | up |
| Bacteroidetes;Bacteroidia;Bacteroidales;Prevotellaceae;Prevotella;2d3104 | 26.1725 | 8.2233 | 2.8464 | 2.8890 | 0.0309 | up |
| Firmicutes;Clostridia;Clostridiales;Lachnospiraceae;Roseburia;2c9d78 | 8.9100 | 6.6761 | 2.3431 | 2.8492 | 0.0342 | up |
| Firmicutes;Clostridia;Clostridiales;NA;NA;d1842b | 10.9324 | 6.9706 | 2.4528 | 2.8419 | 0.0342 | up |
| Firmicutes;Clostridia;Clostridiales;Ruminococcaceae;NA;d3894d | 67.7773 | -4.4769 | 1.5752 | -2.8421 | 0.0342 | down |
| Firmicutes;Erysipelotrichia;Erysipelotrichales;Erysipelotrichaceae;NA;e92e87 | 19.9890 | 7.8350 | 2.7568 | 2.8421 | 0.0342 | up |
| Firmicutes;Negativicutes;Selenomonadales;Veillonellaceae;NA;1e99a0 | 19.1772 | 7.7756 | 2.7510 | 2.8264 | 0.0355 | up |
| Firmicutes;Clostridia;Clostridiales;Ruminococcaceae;Clostridium_IV;9e08de | 10.2543 | -7.0150 | 2.4985 | -2.8077 | 0.0368 | down |
| Firmicutes;Negativicutes;Selenomonadales;Veillonellaceae;NA;7593da | 18.4252 | 7.7182 | 2.7483 | 2.8084 | 0.0368 | up |
| Firmicutes;Clostridia;Clostridiales;Ruminococcaceae;NA;29a33f | 8.9766 | -6.8153 | 2.4411 | -2.7919 | 0.0382 | down |
| Firmicutes;Negativicutes;Selenomonadales;Veillonellaceae;Mitsuokella;c3d1d6 | 408.6157 | 5.9353 | 2.1363 | 2.7783 | 0.0394 | up |
| Firmicutes;Clostridia;Clostridiales;Clostridiaceae_1;Clostridium_sensu_stricto;bda04d | 8.6857 | -6.7688 | 2.4505 | -2.7622 | 0.0406 | down |
| Firmicutes;Clostridia;Clostridiales;NA;NA;ce383e | 17.2919 | -5.6769 | 2.0539 | -2.7639 | 0.0406 | down |
| Firmicutes;Clostridia;Clostridiales;Lachnospiraceae;NA;bc1cc6 | 16.6995 | -7.7140 | 2.7976 | -2.7574 | 0.0407 | down |
| Firmicutes;Negativicutes;Selenomonadales;Veillonellaceae;NA;3f00ec | 16.6644 | 7.5726 | 2.7500 | 2.7537 | 0.0408 | up |
| Firmicutes;Clostridia;Clostridiales;Lachnospiraceae;NA;b09a3a | 10.2351 | 6.8758 | 2.5080 | 2.7416 | 0.0419 | up |
| Bacteroidetes;Bacteroidia;Bacteroidales;Prevotellaceae;Prevotella;f83471 | 180.6926 | -4.4137 | 1.6120 | -2.7380 | 0.0419 | down |
| Firmicutes;Clostridia;Clostridiales;NA;NA;208fd7 | 8.9506 | -6.8145 | 2.5051 | -2.7202 | 0.0434 | down |
| Firmicutes;Clostridia;Clostridiales;Ruminococcaceae;Sporobacter;126e83 | 10.1166 | -6.9903 | 2.5701 | -2.7199 | 0.0434 | down |
| Firmicutes;Clostridia;Clostridiales;Ruminococcaceae;Sporobacter;c01b08 | 9.8921 | -6.9577 | 2.5616 | -2.7162 | 0.0434 | down |
| Firmicutes;Negativicutes;Selenomonadales;Veillonellaceae;Mitsuokella;d83ba4 | 176.3721 | 5.2597 | 1.9443 | 2.7051 | 0.0445 | up |
| Firmicutes;Clostridia;Clostridiales;Lachnospiraceae;NA;471b00 | 13.1270 | 6.2575 | 2.3188 | 2.6986 | 0.0448 | up |
| Firmicutes;Negativicutes;Selenomonadales;Veillonellaceae;Dialister;d8ce6b | 134.3500 | 5.1490 | 1.9095 | 2.6965 | 0.0448 | up |
| Firmicutes;Clostridia;Clostridiales;Lachnospiraceae;Anaerostipes;dd7530 | 7.7596 | 6.4666 | 2.4016 | 2.6926 | 0.0449 | up |
| Firmicutes;Clostridia;Clostridiales;Lachnospiraceae;Clostridium_XlVa;4f2fad | 7.7574 | 6.4775 | 2.4116 | 2.6859 | 0.0454 | up |
| Firmicutes;Bacilli;Lactobacillales;Lactobacillaceae;Lactobacillus;d448ea | 6.5739 | -6.3669 | 2.3839 | -2.6708 | 0.0470 | down |
| Actinobacteria;Actinobacteria;Bifidobacteriales;Bifidobacteriaceae;Bifidobacterium;91b6b2 | 7.2408 | -6.5063 | 2.4407 | -2.6658 | 0.0473 | down |
| Bacteroidetes;Bacteroidia;Bacteroidales;Porphyromonadaceae;NA;5626ad | 12.7042 | -7.3235 | 2.7603 | -2.6531 | 0.0482 | down |
| Bacteroidetes;Flavobacteriia;Flavobacteriales;Flavobacteriaceae;NA;819995 | 12.2505 | -7.2676 | 2.7391 | -2.6533 | 0.0482 | down |
| Spirochaetes;Spirochaetia;Spirochaetales;Spirochaetaceae;Sphaerochaeta;7d4f7b | 11.4272 | -7.1694 | 2.7053 | -2.6502 | 0.0482 | down |
| Firmicutes;Clostridia;Clostridiales;Ruminococcaceae;NA;040104 | 6.1235 | -6.2672 | 2.3720 | -2.6421 | 0.0485 | down |
| Firmicutes;Clostridia;Clostridiales;Ruminococcaceae;NA;b5be61 | 22.4422 | -5.1959 | 1.9650 | -2.6441 | 0.0485 | down |
| Bacteroidetes;Bacteroidia;Bacteroidales;Prevotellaceae;Prevotella;6b87bf | 9.6706 | 6.7776 | 2.5865 | 2.6204 | 0.0491 | up |
| Firmicutes;Bacilli;Lactobacillales;Lactobacillaceae;Lactobacillus;83138f | 6.0952 | -6.2583 | 2.3818 | -2.6276 | 0.0491 | down |
| Firmicutes;Clostridia;Clostridiales;Lachnospiraceae;Dorea;53d431 | 10.7975 | -5.3647 | 2.0431 | -2.6258 | 0.0491 | down |
| Firmicutes;Clostridia;Clostridiales;Ruminococcaceae;NA;474aba | 10.5757 | -6.0367 | 2.2960 | -2.6292 | 0.0491 | down |
| Firmicutes;Clostridia;Clostridiales;Ruminococcaceae;Pseudoflavonifractor;ccdacb | 28.2202 | -5.5219 | 2.0980 | -2.6320 | 0.0491 | down |
| Firmicutes;Negativicutes;Selenomonadales;Acidaminococcaceae;Acidaminococcus;d9ab2e | 50.0187 | 4.5732 | 1.7442 | 2.6220 | 0.0491 | up |
| Firmicutes;NA;NA;NA;NA;5512fa | 6.4624 | -6.3513 | 2.4307 | -2.6129 | 0.0498 | down |

**Supplementary Table 4.** Summary of results of plasma metabolomics analysis. Data represent the mean and standard deviation of three replicates for each treatment, after applying a fold-change threshold of two and false discovery rate (*q)* of 0.1.

| **Group** | | **Metabolite** | **t stat** | **p value** | **-log10 (p)** | **FDR-adjusted**  **p value** |
| --- | --- | --- | --- | --- | --- | --- |
| **Amino acids and metabolism derivatives** | **Amino Acids** | Arginine | -2.1696 | 0.095855 | 1.0184 | 0.23105 |
|  |  | Methionine | -2.4084 | 0.073684 | 1.1326 | 0.23105 |
|  |  | Phenylalanine | 3.9497 | 0.016822 | 1.7741 | 0.13217 |
|  |  | Tryptophan | 2.7394 | 0.051937 | 1.2845 | 0.23105 |
|  |  | Tyramine | 1.9323 | 0.12548 | 0.90141 | 0.23692 |
|  |  | Tyrosine | 8.7065 | 0.0009583 | 3.0185 | **0.041499** |
|  |  | Homocysteine | 7.7289 | 0.001509 | 2.8213 | **0.041499** |
|  | **Glycine Derivative** | Dimethylglycine | -0.40567 | 0.70575 | 0.15135 | 0.74647 |
|  | **Homocysteine Metabolite** | S-Adenosyl-L-homocysteine | 1.1637 | 0.30923 | 0.50972 | 0.3517 |
|  | **Homocysteine Metabolite** | Homocysteic acid | 3.3662 | 0.028143 | 1.5506 | 0.19348 |
|  | **Methionine Metabolite** | S-adenosylmethionine | -2.0905 | 0.10477 | 0.97975 | 0.23105 |
|  | **Phenylalanine Metabolite** | Phenylethylamine | -1.4112 | 0.23102 | 0.63636 | 0.30069 |
|  | **Tryptophan Metabolites** | 3-Hydroxyanthranilic acid | -1.3903 | 0.23679 | 0.62564 | 0.30069 |
|  |  | 5-hydroxytryptamine (Serotonin) | -2.1304 | 0.10016 | 0.9993 | 0.23105 |
|  |  | Kynurenic acid | -0.12101 | 0.90952 | 0.041187 | 0.90952 |
|  |  | Kynurenine | 2.7227 | 0.05284 | 1.277 | 0.23105 |
|  |  | Tryptamine | -1.2859 | 0.26787 | 0.57207 | 0.31347 |
| **Short chain fatty acids** | | Formic acid | -2.0959 | 0.10414 | 0.9824 | 0.23105 |
|  |  | Isobutyric Acid | -2.0022 | 0.11582 | 0.93621 | 0.23593 |
|  |  | Isovaleric acid | -1.9067 | 0.12923 | 0.88864 | 0.23692 |
|  |  | 3-methyl valeric acid | -1.4803 | 0.21291 | 0.67181 | 0.30026 |
|  |  | 4-methyl valeric acid | -1.5705 | 0.1914 | 0.71807 | 0.29033 |
|  |  | Acetic Acid | -5.5113 | 0.0052888 | 2.2766 | **0.058176** |
|  |  | Butyric acid | -1.8382 | 0.13989 | 0.85422 | 0.24663 |
|  |  | Propionic Acid | -1.5307 | 0.20059 | 0.69768 | 0.29033 |
|  |  | Valeric acid | -1.9159 | 0.12788 | 0.89321 | 0.23692 |
| **Medium chain fatty acids** | | Heptanoic acid | -1.8163 | 0.14349 | 0.84317 | 0.24663 |
|  |  | Hexanoic acid | -1.6046 | 0.18386 | 0.73552 | 0.28892 |
| **Uremic Solutes and Precursors** | **Uremic Solutes** | trimethylamine-N-oxide | 6.5545 | 0.0028019 | 2.5526 | **0.049286** |
|  |  | indoxyl sulphate | 1.377 | 0.24055 | 0.61879 | 0.30069 |
|  |  | p-cresol glucuronide | 1.026 | 0.36288 | 0.44024 | 0.39916 |
|  |  | p-cresyl sulphate | 0.9969 | 0.37523 | 0.4257 | 0.40466 |
|  |  | Indole | -1.3995 | 0.23424 | 0.63033 | 0.30069 |
|  |  | p-cresol | 1.5522 | 0.19556 | 0.70871 | 0.29033 |
|  | **TMAO Precursors** | trimethylamine | 2.2448 | 0.088154 | 1.0548 | 0.23105 |
|  |  | Betaine | -2.2978 | 0.083138 | 1.0802 | 0.23105 |
|  |  | Carnitine | 2.7874 | 0.049445 | 1.3059 | 0.23105 |
|  |  | Choline | -2.2986 | 0.083067 | 1.0806 | 0.23105 |
|  | **Choline metabolites** | Glycerophosphocholine | -2.3035 | 0.08262 | 1.0829 | 0.23105 |
|  |  | Phosphocholine | -2.6543 | 0.056727 | 1.2462 | 0.23105 |
| **Creatine and metabolites** | | Creatine | -1.2972 | 0.26433 | 0.57785 | 0.31347 |
|  |  | Creatinine | 2.0869 | 0.1052 | 0.978 | 0.23105 |
|  |  | Phosphocreatine | -1.6144 | 0.18175 | 0.74054 | 0.28892 |
| **Ketosis markers** | | 2-OH-Glutarate | 0.34883 | 0.74481 | 0.12796 | 0.7586 |
|  |  | Acetoacetate | 0.35726 | 0.73895 | 0.13139 | 0.7586 |
|  |  | Glyoxalate | 2.2571 | 0.086963 | 1.0607 | 0.23105 |
|  |  | Hydroxybutyric acid | 1.389 | 0.23717 | 0.62493 | 0.30069 |
|  |  | Methylglyoxal | 2.2585 | 0.086823 | 1.0614 | 0.23105 |
| **Antioxidants** | | Tyrosol | -1.2954 | 0.26489 | 0.57693 | 0.31347 |
|  |  | oxidized glutathione | 1.6064 | 0.18345 | 0.73648 | 0.28892 |
|  |  | glutathione | -2.0537 | 0.10923 | 0.96168 | 0.23105 |
| **Other** | **Neurotransmitter precursor** | Myo-Inositol | -1.1524 | 0.31333 | 0.504 | 0.3517 |
|  | **Pro-inflammatory marker** | Neopterin | 6.132 | 0.0035844 | 2.4456 | **0.049286** |
|  |  | 2,3-Dihydroxybenzoic acid | 4.1058 | 0.014783 | 1.8302 | 0.13217 |


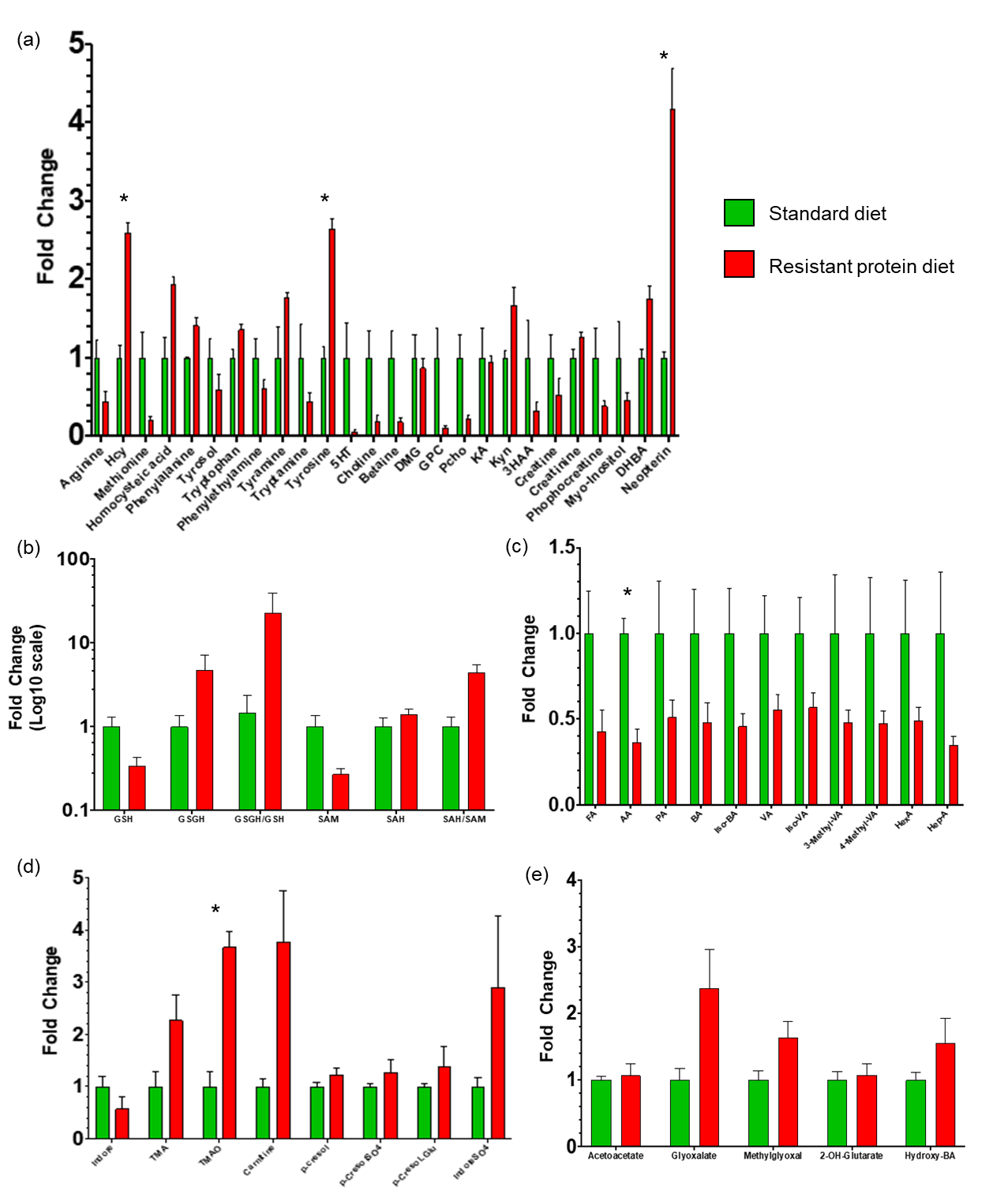


**Supplementary Figure 1**. Mean fold-change results of plasma biomarkers for (a) selected general metabolites, (b) oxidative stress regulation, (c) short-chain fatty acids, (d) uremic solutes, and (e) ketosis. Results represent the mean and standard error of the mean (n=3). Statistical differences between groups are designated * *p* < 0.05.
Hcy, homocysteine; 5HT, 5-hydroxytryptamine (serotonin); DMG, dimethylglycine; GPC, glycerophosphocholine; PCho, phosphocholine; KA, kynurenic acid; Kyn, kynurenine; 3HAA, 3-hydroxyanthranilic acid; DHBA, dihydroxybenzoic acid; GSH, glutathione; GSGH, oxidized glutathione; SAM, S-adenosylmethionine; SAH, S-andenosylhomocysteine; FA, formic acid; AA, acetic acid; PA, propionic acid; BA, butyric acid; VA, valeric acid; HexA, hexanoic acid; HepA heptanoic acid; TMA, trimethylamine; TMAO, trimethylamine-n-oxide.


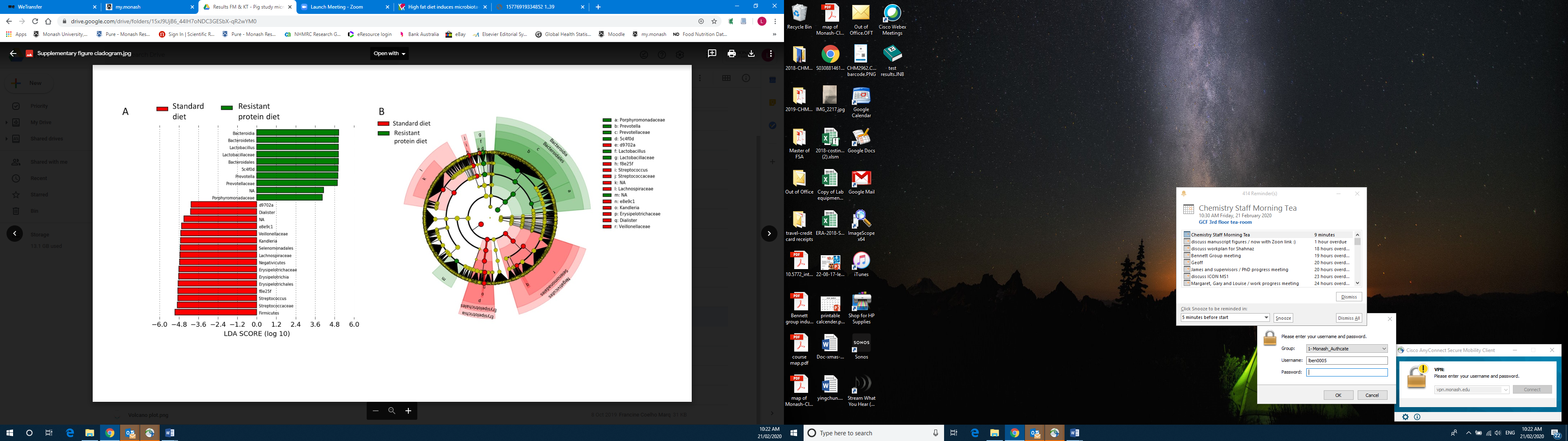


**Supplementary Figure 2.** Abundant taxa based on diet and their effect sizes. (a) Taxonomic differences with a score >4 ranked by effect size according to diet and, (b) Cladogram showing phylogenetic tree of taxonomic differences between standard and resistant protein diets.
